# Assessing the Evidence for Causal Associations Between Body Mass Index, C-Reactive Protein, Depression, and Reported Trauma Using Mendelian Randomization

**DOI:** 10.1016/j.bpsgos.2022.01.003

**Published:** 2022-01-28

**Authors:** Alish B. Palmos, Christopher Hübel, Kai Xiang Lim, Avina K. Hunjan, Jonathan R.I. Coleman, Gerome Breen

**Affiliations:** a Social, Genetic and Developmental Psychiatry Centre, Institute of Psychiatry, Psychology & Neuroscience, King’s College London, London, United Kingdom; b UK National Institute for Health Research Biomedical Research Centre for Mental Health, South London and Maudsley Hospital, London, United Kingdom; c National Centre for Register-based Research, Department of Economics and Business Economics, Aarhus University, Aarhus, Denmark

**Keywords:** Body mass index, Causation, Depression, Genetics, Inflammation, Mendelian randomization, Trauma

## Abstract

**Background:**

Traumatic experiences are described as the strongest predictors of major depressive disorder (MDD), with inflammation potentially mediating the association between trauma and symptom onset. However, several studies indicate that body mass index (BMI) exerts a large confounding effect on both inflammation and MDD.

**Methods:**

First, we sought to replicate previously reported associations between these traits in a large subset of the UK Biobank, using regression models with C-reactive protein (CRP) and MDD and as the outcome variables in 113,481 and 30,137 individuals, respectively. Second, we ran bidirectional Mendelian randomization analyses between these traits to establish a potential causal framework between BMI, MDD, reported childhood trauma, and inflammation.

**Results:**

Our phenotypic analyses revealed no association between CRP and MDD but did suggest a strong effect of BMI and reported trauma on both CRP (BMI: β = 0.43, 95% CI = 0.43–0.43, *p* ≤ .001; childhood trauma: β = 0.02, 95% CI = 0.00–0.03, *p* = .006) and MDD (BMI: odds ratio [OR] = 1.16, 95% CI = 1.14–1.19, *p* ≤ .001; childhood trauma: OR = 1.99, 95% CI = 1.88–2.11, *p* ≤ .001). Our Mendelian randomization analyses confirmed a lack of causal relationship between CRP and MDD but showed evidence consistent with a strong causal influence of higher BMI on increased CRP (β = 0.37, 95% CI = 0.36–0.39, *p* ≤ .001) and a bidirectional influence between reported trauma and MDD (OR trauma-MDD = 1.75, 95% CI = 1.49–2.07, *p* ≤ .001; OR MDD-trauma = 1.22, 95% CI = 1.18–1.27, *p* ≤ .001).

**Conclusions:**

Our findings highlight the importance of controlling for both BMI and trauma when studying MDD in the context of inflammation. They also suggest that the experience of traumatic events can increase the risk for MDD and that MDD can increase the experience of traumatic events.

Major depressive disorder (MDD) is arguably the single largest contributor to global disability (1); however, it is not fully understood how environmental, developmental, and genetic risks give rise to MDD. Numerous causal mechanisms have been proposed, with some studies suggesting that MDD may manifest as a result of aberrant immune functioning in the body (2,3). In this hypothesis, overactivation of inflammatory pathways leads to a systemic increase in immune modulators known as cytokines, which have been associated with psychiatric symptoms in both humans and animal models (2,4). A subtype of MDD, associated with raised inflammatory markers, may arise as a consequence of childhood trauma (5). Indeed, childhood trauma is associated with increases in proinflammatory markers, such as C-reactive protein (CRP), interleukin 6 (IL-6), and tumor necrosis factor α, and these markers are, in turn, associated with symptoms of MDD, thus supporting the inflammatory hypothesis of MDD (5, 6, 7, 8). In addition, a recent genetic study reported a greater genetic heritability of MDD in participants reporting trauma compared with unexposed cases, supporting the notion that reported trauma is a strong predictor of MDD risk (9). The authors suggested a greater combined effect of the variants associated with MDD in those reporting trauma compared with those who do not report trauma, possibly given that exposure to traumatic events might amplify genetic influences on MDD compared with the absence of trauma. Taken together, these findings suggest that inflammation may potentially mediate the effect of trauma on MDD risk.

However, results are somewhat inconsistent. In a sample of trauma-exposed and trauma-unexposed individuals, of 42 inflammatory markers, none were associated with clinically diagnosed MDD, but a possible confounding effect of body mass index (BMI) on CRP and IL-6 levels was detected (10). Raised circulating proinflammatory cytokines have also been frequently associated with higher BMI, smoking, and more sedentary lifestyles, all of which are common in people with a psychiatric diagnosis (11,12). To overcome the effect of these confounding factors, we carried out previous research using genetic risk scores for MDD as proxies for MDD in a healthy population, detecting no significant associations between polygenic risk for MDD and inflammatory markers (13). However, we did show an association between genetic risk scores for higher BMI and inflammatory markers IL-6 and CRP. This suggests a complex network of effects between BMI, MDD, CRP, and trauma that needs further investigation. More specifically, there is a need to understand the independent effects of trauma on MDD and CRP and whether BMI is independently associated with all or any of these traits, confirming its role as a major confounding factor.

One applicable method to disentangle the relationship between these traits is Mendelian randomization (MR). MR allows estimation of putative causal effects of an exposure on a disease or a disease-related trait with a reduced bias of environmental confounding effects. Recent advancements in MR methods allow use of summary data of genetic associations obtained from genome-wide association studies (GWASs), linking potentially modifiable risk factors to disease outcomes (14).

To understand the causal interrelationships among BMI, trauma, inflammation, and MDD, we therefore carried out a two-stage analysis. First, we attempted to replicate the phenotypic associations between BMI, MDD, CRP, and reported trauma, as reported in our previous studies using smaller cohorts (10,13,15), this time using a much larger sample made up of the UK Biobank (13). Second, we used MR to investigate potential causal relationships among these traits (16, 17, 18).

## Methods and Materials

### Phenotypic Associations

To confirm previously reported associations between MDD and circulating proinflammatory markers, we used the UK Biobank, a large prospective cohort study that assesses a wide range of health-related measures (including BMI and CRP) as well as genome-wide genetic variation data in approximately 500,000 individuals (19). This includes a common mental health disorders questionnaire and 16 items that assess lifetime traumatic life events (20). After quality control, 113,481 people had genetic, BMI, CRP, and reported trauma data available, of which 30,137 had also taken part in the mental health survey. See Table 1 for a full breakdown of the samples included in our phenotypic models.

**Table 1:** UK Biobank Samples (*N* = 113,481) Included in Phenotypic Analyses

Characteristic	Value
Age, Years, Mean (SD)	56.0 (7.7)
Sex, Female, *n* (%)	63,209 (55.7%)
Smoking Status, *n* (%)	
No answer	205 (0.2%)
Never	65,061 (57.3%)
Previous	40,216 (35.4%)
Current	7999 (7.0%)
Body Mass Index, Mean (SD)	26.6 (4.4)
Childhood Trauma, *n* (%)	26,746 (23.6%)
Adulthood Trauma, *n* (%)	20,946 (18.5%)
Physical Trauma, *n* (%)	4459 (3.9%)
Lifetime Depression, *n* (%)	8546 (7.6%)

This table details the sample demographics used in the phenotypic analyses, with C-reactive protein and lifetime depression as the outcomes of interest.

Our outcomes variables were MDD and CRP. Independent variables included MDD, CRP, BMI, childhood trauma, adulthood trauma, and physical trauma, alongside polygenic scores for MDD and BMI due to their reported associations with MDD and CRP (13,21). See the Supplement for details on how these phenotypes were constructed. Note that given that this step was a replication analysis, we constructed every single variable in such a way that they accurately resembled previous study measures. We therefore did not construct polygenic scores for traits that were not part of the replication step.

### Mendelian Randomization

In the second part of the study, we used the generalized summary data–based MR (GSMR) method to investigate potential genetic predictions between our four traits of interest (CRP, MDD, BMI, and trauma) (22). Each trait was analyzed as an exposure and an outcome, resulting in a total of 12 MR analyses. Any significant results from GSMR analyses were put through sensitivity analyses using additional MR methods (16,18,23).

GSMR analyses were performed using the largest available GWAS for each trait. In brief, we used the latest Psychiatric Genomic Consortium MDD GWAS (with 23andMe) (24,25), the latest CHARGE Consortium CRP GWAS (26), the largest GIANT Consortium BMI GWAS (a meta-analysis between the GIANT BMI GWAS and the UK Biobank BMI GWAS) (27), and the largest GWAS of childhood trauma (28). More information regarding the publicly available GWAS summary statistics can be found in the Supplement. Given the sensitivity of trauma research, we should note that we are not discussing a genetic risk for being exposed to childhood trauma, but rather a complex phenotype encompassing the genetic propensity for behaviors, personality types, and cognitive factors, which influence the reporting of childhood traumatic events (29, 30, 31).

### Statistical Analyses

#### Phenotypic Associations

All statistical analyses were carried out using R version 3.6.0. Phenotypic associations were estimated to replicate previous findings (13). Each independent variable was independently tested using either a logistic model (in the case of the binary lifetime depression outcome [referred to as MDD]) or a linear model (in the case of the continuous CRP outcome). Each model controlled for age, sex, the first six genomic principal components, 21 assessment center covariates, 105 batch covariates, fasting time, smoking status, and BMI (except when BMI was the predictor of interest). BMI was not scaled for these analyses.

#### Mendelian Randomization Analyses

GSMR analyses were carried out to establish potential causal effects between our traits of interest. GSMR first identifies genome-wide significant genetic variants between the exposure, the outcome, and a reference panel and then filters out genetic variants with missing values or mismatched alleles. Note that GSMR is a powerful method because it accounts for sampling variance for each genetic variant and the linkage disequilibrium (LD) among the variants using a reference panel, thus allowing the use of partially overlapping samples. We used the 1000 Genomes Project LD reference panel for all analyses, as described previously (32). GSMR then removes variants with large differences in allele frequency among the GWAS summary data and the reference panel and filters out pleiotropic single nucleotide polymorphisms using the HEIDI outlier method (22). The remaining genetic variants are used in the bidirectional MR analysis between the exposure and the outcome of interest. Single nucleotide polymorphisms were obtained below the *p* < 5 × 10^−8^ for all GWASs. The LD *r*^2^ was set to 0.05 and the HEIDI threshold was set to 0.01 for all analyses, as suggested by the authors of this method. Full details on the number of genetic variants used as instruments and the other parameter thresholds used in our GSMR analysis can be found in the Supplement. Estimates with binary phenotypes were converted to a liability scale using previously reported methods (33).

#### Mendelian Randomization Sensitivity Analyses

As a sensitivity analysis for GSMR, we performed MR using four robust MR methods plus inverse-variance weighted method, via the TwoSampleMR package in R (34). The TwoSampleMR package implements MR-Egger, weighted-median, inverse-variance weighted, simple mode, and weighted mode methods. These have previously been described as robust MR methods, capable of detecting pleiotropy between genetic instruments and evaluating the impact of weak genetic instruments (16, 17, 18). In addition, we used multitrait-based conditional and joint analysis to condition MDD, CRP, and reported trauma on BMI, which adjusts these three traits for genetic association with BMI. We then reran all GSMR analyses to investigate the degree to which BMI is affecting the causal pathways. We also performed multivariable MR (MVMR) analyses using the MVMR package (35) with MDD as the outcome and BMI, CRP, and reported trauma as joint exposures. MVMR allows us to estimate the joint effect of each exposure on the outcome (35). In addition, we calculated *F* statistics and *I*^2^ statistics for all our traits to investigate weak genetic instrument bias (36). Next, we isolated *cis*-CRP genetic variants to test for the association with MDD and reported trauma, as carried out in previous studies (37, 38, 39). We identified *cis*-CRP genetic variants using LD-link (40) as those within the CRP coding region in a European population (39). We then ran GSMR analyses to test for the genetic effect of CRP on childhood trauma and MDD at five different clumping thresholds (*R*^2^ < 0.05, *R*^2^ < 0.2, *R*^2^ < 0.4, *R*^2^ < 0.6, *R*^2^ < 0.8). This was carried out to compare our results with other studies using various clumping parameters (37, 38, 39). We also performed bidirectional MR-CAUSE analyses between all our traits. MR-CAUSE is a tool able to identify patterns consistent with causal effects while accounting for pleiotropic effects, with a high degree of control for false positive effects (41). If the causal model is not the best fit, MR-CAUSE suggests a sharing model as the best fit.

## Results

### Phenotypic Models in the UK Biobank

In the first part of the study, we sought to replicate previous findings, as reported in smaller cohorts. Linear models were compared between all exposures of interest and two outcomes, a binary (yes/no) lifetime depression measure (MDD) and a continuous log-transformed circulating CRP measure. See Tables S1 and S2 for more details.

#### Phenotypic Associations With Depression as the Outcome

**Figure 1:**
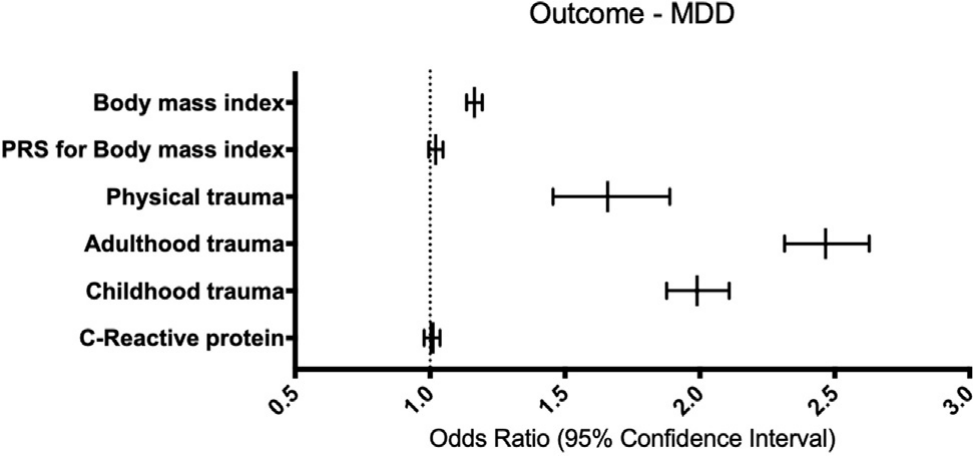
Phenotypic associations with probable lifetime MDD as the outcome in the UK Biobank. This figure represents a summary of six generalized linear models with MDD as the outcome and each trait of interest as an independent variable. Each model was controlled for age, sex, six population covariates, 21 assessment center covariates, 105 batch covariates, fasting time, smoking status, and BMI (with the exception of BMI being the independent variable of interest). Only the traits of interest are shown on the y-axis, with the odds of each predictor on the outcome shown on the x-axis (as an odds ratio plotted on a linear scale). Error bars represent a 95% confidence interval. The dashed line represents an odds ratio of 1, equaling no effect. BMI, physical trauma, adulthood trauma, and childhood trauma were significantly associated with higher odds of lifetime MDD after multiple testing correction (*p*_Bonferroni_ = .008). BMI, body mass index; MDD, major depressive disorder; PRS, polygenic risk score.

Generalized linear models with a binary outcome for MDD indicate that BMI is significantly associated with higher odds for MDD (odds ratio [OR] = 1.17, 95% CI = 1.14–1.20, *p* ≤ .001). Likewise, reported childhood trauma (OR = 1.99, 95% CI = 1.88–2.11, *p* ≤ .001), reported adulthood trauma (OR = 2.47, 95% CI = 2.31–2.63, *p* ≤ .001), and reported physical trauma (OR = 1.66, 95% CI = 1.46–1.89, *p* ≤ .001) are all significantly associated with higher odds for MDD. These results remain significant after Bonferroni correction (*p*_Bonferroni_ = .008). However, CRP and polygenic score for BMI are not significantly associated with MDD (Figure 1).

#### Phenotypic Associations With CRP as the Outcome

**Figure 2:**
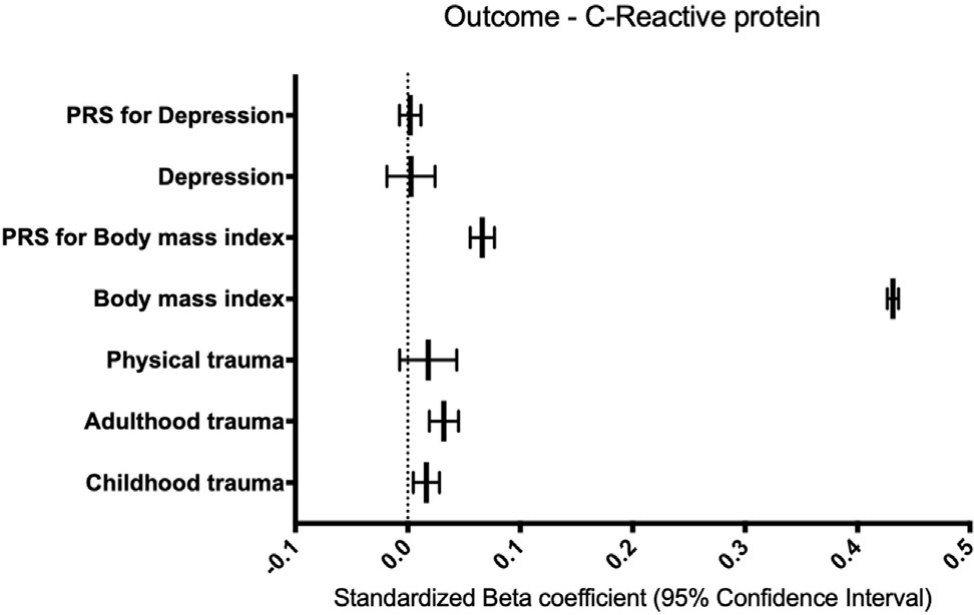
Phenotypic associations with CRP as the outcome in the UK Biobank. This figure represents a summary of seven linear models with CRP as the outcome and each trait of interest as an independent variable. Each model was controlled for age, sex, six population covariates, 21 assessment center covariates, 105 batch covariates, fasting time, smoking status, and body mass index (with the exception of body mass index being the independent variable of interest). Only the traits of interest are shown on the y-axis, with the standardized β of each predictor on the outcome shown on the x-axis. Error bars represent a 95% confidence interval. The dashed line represents a standardized β of zero. Body mass index, PRS for body mass index, adulthood trauma, and childhood trauma were significantly associated with increased circulating CRP levels after multiple testing correction (*p*_Bonferroni_ = .008). CRP, C-reactive protein; PRS, polygenic risk score.

Linear models with log-transformed circulating CRP level as the outcome indicate that BMI (β = 0.44, 95% CI = 0.43–0.44, *p* ≤ .001) and polygenic risk scores (PRSs) for BMI (β = 0.07, 95% CI = 0.06–0.08, *p* ≤ .001) are significantly associated with circulating CRP levels. These results remain significant after Bonferroni correction (*p* = .007). MDD and a PRS for MDD were not significantly associated with circulating CRP levels. In addition, reported childhood trauma (β = 0.02, 95% CI = 0.01–0.03, *p* = .006) and reported adulthood trauma (β = 0.03, 95% CI = 0.02–0.04, *p* ≤ .001), but not reported physical trauma, were significantly associated with circulating CRP levels. These results remain significant after Bonferroni correction (*p*_Bonferroni_ = .008) (Figure 2).

### Mendelian Randomization Results

In the second part of the study, we carried out a series of bidirectional MR analyses with CRP, MDD, BMI, and childhood trauma to develop a causal framework for the etiology of MDD. Each trait was analyzed as an exposure and as an outcome, resulting in a total of 12 MR analyses. See Table S3 and Figures S1–S12 for more details. Note that MR investigates the genetic component of an exposure predicting an outcome with pleiotropic effects removed, which can suggest evidence consistent with a causal relationship. In the results, we refer to this as a genetic prediction, as suggested by Burgess *et al.* (18).

#### CRP as the Outcome

**Figure 3:**
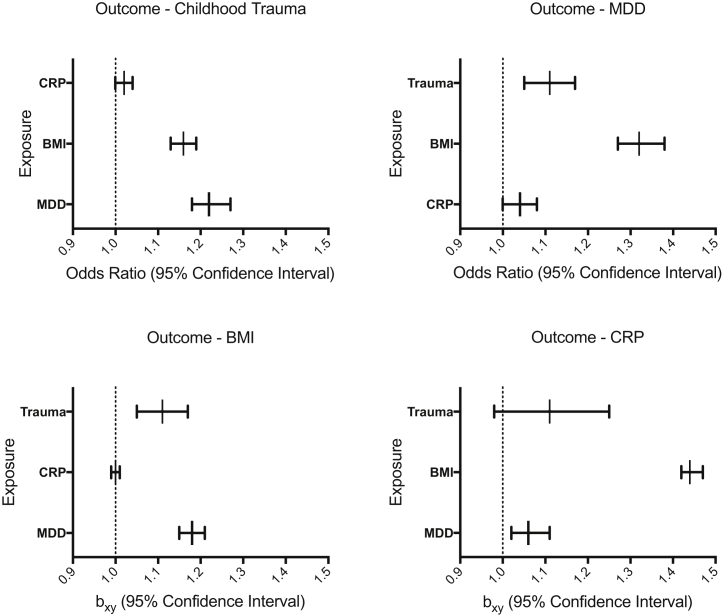
All generalized summary-data–based Mendelian randomization results. This figure represents a summary of three bidirectional generalized summary-data–based Mendelian randomization analyses involving four traits (12 analyses in total). The charts are split by the exposure of interest. Dots represent effect sizes (as measured by odds ratios) on the liability scale of the disorders of risk factors on traits (childhood trauma and MDD) and effect sizes (as measured by β, b_xy_) on the liability scale of the disorders of risk factors on traits (BMI and CRP). Each outcome is labeled on the y-axis and the strength of each exposure on the outcome displayed on the x-axis (as an odds ratio or β, plotted on a linear scale). Error bars represent 95% confidence intervals. BMI as the exposure was significantly associated with all three traits, reported trauma as the exposure was significantly associated with MDD, and MDD as the exposure was significantly associated with BMI and reported trauma after multiple testing correction (*p*_Bonferroni_ = .004). BMI, body mass index; CRP, C-reactive protein; MDD, major depressive disorder.

MR analyses with CRP as the outcome indicate that BMI genetically predicts higher CRP (β = 0.37, 95% CI = 0.36–0.39, *p* ≤ .001), whereby a 1-kg/m^2^ increase in BMI is associated with a 0.37-mg/L increase in CRP. This finding remains significant after Bonferroni correction (*p*_Bonferroni_ = .004). MDD was not found to genetically predict CRP (β = 0.06, 95% CI = 0.02–0.1, *p* = .006), and childhood trauma was not found to genetically predict CRP (β = 0.103, 95% CI = 0.02–0.22, *p* = .09) (Figure 3).

#### Depression as the Outcome

MR analyses with MDD as the outcome indicate that BMI genetically predicts higher odds of MDD (OR = 1.32, 95% CI = 1.27–1.38, *p* ≤ .001), whereby a 1-kg/m^2^ increase in BMI is associated with 32% higher odds of MDD, and childhood trauma genetically predicts higher odds of MDD (OR = 1.75, 95% CI = 1.49–2.07, *p* ≤ .001), whereby reporting childhood trauma is associated with 75% higher odds of MDD. This remains significant after Bonferroni correction (*p*_Bonferroni_ = .004) (Figure 3).

#### BMI as the Outcome

MR analyses with BMI as the outcome indicate that MDD genetically predicts a moderate increase in BMI (β = 0.17, 95% CI = 0.14–0.19, *p* ≤ .001), whereby MDD is associated with a 0.17 kg/m^2^ higher BMI, and childhood trauma genetically predicts higher BMI (β = 0.1, 95% CI = 0.05–0.15, *p* ≤ .001), whereby childhood trauma is associated with a 0.1-kg/m^2^ higher BMI. These findings were significant after Bonferroni correction (*p*_Bonferroni_ = .004). CRP was not found to genetically predict BMI (OR = 1.00, 95% CI = 0.99–1.01, *p* = .73) (Figure 3).

#### Childhood Trauma as the Outcome

MR analyses with childhood trauma as the outcome indicate that MDD genetically predicts higher odds of childhood trauma (OR = 1.22, 95% CI = 1.18–1.27, *p* ≤ .001), whereby MDD is associated with 22% higher odds of reporting a childhood trauma, and BMI genetically predicts higher odds of childhood trauma (OR = 1.16, 95% CI = 1.13–1.19, *p* ≤ .001), whereby a 1-kg/m^2^ increase in BMI is associated with 16% higher odds of reporting a childhood trauma. These findings remain significant after Bonferroni multiple testing correction (*p*_Bonferroni_ = .004). CRP was not found to genetically predict childhood trauma (OR = 1.02, 95% CI = 1.00–1.04, *p* = .07) (Figure 3).

### Mendelian Randomization Sensitivity Analyses

Any significant results from GSMR analyses were subjected to sensitivity analyses using multiple MR methods (16, 17, 18). Results from sensitivity analyses that align with the findings from GSMR using at least three of the five sensitivity methods are BMI (exposure) genetically predicting MDD (outcome), BMI (exposure) genetically predicting CRP (outcome), childhood trauma (exposure) genetically predicting MDD (outcome), and MDD (exposure) genetically predicting childhood trauma (outcome). Other analyses did not reach significance in three of the five methods. See the Supplement for more details (Tables S4–S15 and Figures S13–S24).

In addition, we isolated *cis*-CRP genetic variants to test for the association with reported trauma and MDD, as carried out in previous studies (37, 38, 39). Our findings are mixed, suggesting that *cis*-CRP genetic variants mildly genetically predict higher odds of MDD at *R*^2^ < 0.8 (OR = 1.03, 95% CI = 1.00–1.05, *p* = .02), although they do not at the advised threshold of *R*^2^ < 0.05 (OR = 1.02, 95% CI = 0.97–1.06, *p* = .5). See the Supplement for more details (Table S16).

We also reran GSMR with all traits conditioned on BMI, using multitrait-based conditional and joint analysis (22). This method enables the genetic effect of a risk factor on an outcome variable to be estimated while controlling for another risk factor. Analyses revealed that the bidirectional relationship between MDD and reported trauma remains significant after conditioning on BMI. See the Supplement for full details of these sensitivity analyses (Figure S25). In addition to this, we performed MVMR analyses to understand the joint exposure effect of BMI, CRP, and reported trauma on MDD. Our results indicate the strongest direct effect of reported trauma (OR = 1.57, 95% CI = 1.56–1.58), followed by a direct effect of BMI (OR = 1.11, 95% CI = 1.10–1.12) and a weak negative direct effect of CRP (OR = 0.98, 95% CI= 0.98–0.99) on MDD. See the Supplement for a full table of results (Table S17).

We also calculated *F* statistics and *I*^2^ statistics for all our traits. The *F* statistic was calculated to investigate weak genetic instrument bias, and our findings indicate relative strength of all genetic instruments using the *F* statistic method (*F* > 30), as described in previous studies (36,42,43). The *I*^2^ statistic was calculated as an indicator of the strength of the NOME (NO Measurement Error) violation for MR-Egger (44) and our findings indicate low estimates for reported trauma and MDD, suggesting that MR-Egger estimates for when reported trauma and MDD are exposures of interest should be interpreted with caution. See the Supplement for a full table of results (Table S18).

We also ran MR-CAUSE analyses between our traits to understand which associations are likely to be driven by causal effects and which associations are likely to be driven by shared, pleiotropic effects. Our findings suggest that there is no bidirectional association between CRP and MDD, consistent with our GSMR analyses. Our findings also suggest that the reported trauma effect on MDD is best described by a shared model, whereas the MDD effect on reported trauma is best described by a causal model. This supports the effect of MDD on reported trauma as one of our most robust findings with GSMR and suggests that another process may be associated with the effect of reported trauma on MDD. Our findings also suggest that the effect of BMI on MDD is best described by a shared model, whereas the effect of MDD on BMI is best described by a causal model. Again, this supports the strong effect of MDD on BMI as reported by GSMR and suggests that another process may be associated with the effect of BMI on MDD. See the Supplement for more details (Table S19).

## Discussion

This study was designed with two aims in mind. The first aim was to replicate previous findings regarding inflammation, MDD, BMI, and reported trauma. Many studies focusing on these traits are based on small samples; thus, our goal was to model MDD and CRP as outcome variables in a large sample. We sought to determine whether MDD, BMI, and reported trauma are associated with higher CRP concentrations or the odds of having lifetime MDD in 113,481 and 30,137 individuals of the UK Biobank, respectively. In addition, reported trauma, BMI, and PRSs for MDD and BMI were tested for associations with CRP and MDD (for BMI only) due to previously reported associations (13).

Our analyses reveal no phenotypic association between CRP and MDD. However, they do reveal significant associations of childhood trauma, adulthood trauma, and physical trauma with BMI and MDD. Specifically, the odds of lifetime MDD are found to be higher in the presence of higher BMI, reported childhood trauma, adulthood trauma, and physical trauma. Analyses also reveal that BMI, polygenic risk for higher BMI, childhood trauma, and adulthood trauma are associated with higher concentrations of CRP.

The absence of a phenotypic association between CRP and MDD confirms previous findings (15) and suggests that controlling for confounding factors such as BMI may remove significant associations reported in other studies (11,15,45,46). A significant association of trauma with both CRP and MDD is in line with previous findings suggesting that trauma is associated with inflammation and MDD (2,5,47). Finally, a significant association between a PRS for higher BMI and CRP, but not between a PRS for MDD and CRP, replicates our previous report that showed the same effect in a smaller sample (13).

The second aim of this study was to establish potential causal paths between our traits of interest using MR. Our findings reveal that after multiple testing correction, MDD and CRP do not genetically predict one another. This finding is supported by MR-CAUSE analyses. This is in line with our phenotypic findings but is contrary to many studies reporting CRP as being associated with MDD. Although many of these studies control for BMI, they often do not control for both BMI and trauma (48, 49, 50). Our results suggest that both BMI and trauma are causally associated with MDD, which may explain why our findings differ from others. We should note that previous studies have shown that other proinflammatory cytokines such as IL-6, which were not investigated in this study, are associated with MDD (51). In addition, larger studies able to study MDD subtypes are reporting elevated CRP levels in patients with atypical MDD or those who have suicidal tendencies (52). It is also important to note that MVMR analyses revealed a small but significant negative effect of CRP on MDD, which differs from the positive (although nonsignificant) effect seen in our GSMR analyses.

While we show a phenotypic association between childhood trauma and CRP, a putative causal relationship was not supported by our MR analyses. The lack of a putative causal association is somewhat surprising, given that our phenotypic associations, alongside previous studies, demonstrate associations between different trauma types and inflammation (5,7,47). We used the latest and most powered childhood trauma GWAS to date to obtain enough genetic instruments to carry out MR. However, we cannot rule out the association between other types of traumas and CRP. Indeed Carvalho *et al.* (53) found an association between posttraumatic stress disorder and CRP, which suggests that clinically diagnosed trauma, as in the case of posttraumatic stress disorder, may have a differential causal association with CRP. Further research using well-powered GWASs of trauma subtypes may provide insights regarding this issue. Note that several studies have suggested that *cis*-CRP genetic instruments may be associated with MDD (37, 38, 39), although our sensitivity analyses suggest that this is only the case when using abnormal clumping parameters (*R*^2^ < 0.8). Larger GWASs on CRP and other inflammatory markers may provide more genetic instruments to further elucidate this relationship.

Our phenotypic and MR results suggest a significant bidirectional association between childhood trauma and MDD. Childhood trauma has been robustly associated with MDD (53, 54, 55, 56), and our findings confirm this using MR methods, demonstrating that genetic components of reporting childhood trauma are associated with 75% higher odds of reporting MDD. Note that we are discussing the genetic propensity to interpret a stressful life event as traumatic, and this tendency may have considerable interindividual differences (9). Although traumatic life events are associated with an increased risk of MDD (57), our study shows that there may be a causal path between reporting childhood traumatic life events and MDD. The opposite effect was also significant, although not as strong, with MDD resulting in 22% higher odds of reporting a childhood trauma. This novel finding may partially explain why people who develop MDD may experience and interpret certain life events as traumatic (58). Note that MR-CAUSE analyses indicate that the effect of reported trauma on MDD are best described by a shared model, suggesting another process that may influence both traits, whereas the effect of MDD on reported trauma is best described by a causal model. Nevertheless, MVMR analyses suggest that reported trauma has the strongest positive effect on MDD when measured in conjunction with CRP and BMI.

**Figure 4:**
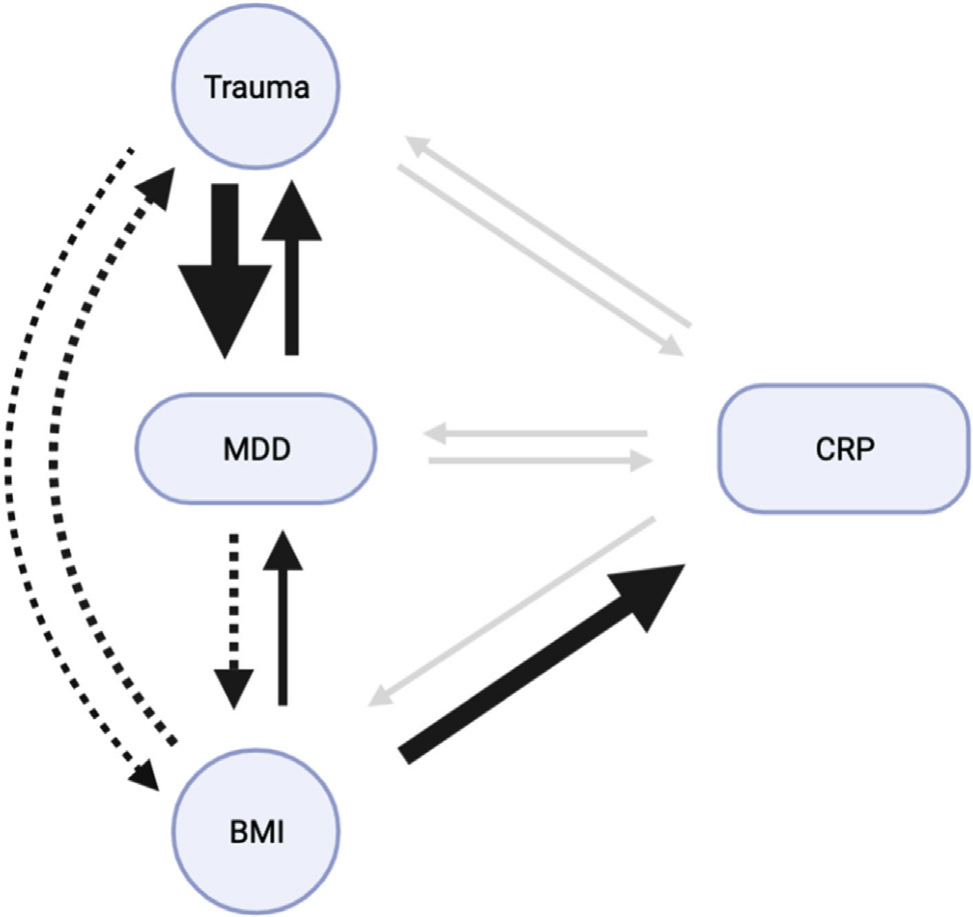
A graphical representation of MR results between our traits of interest using GSMR, displayed using causal paths and their effect sizes. This diagram represents the causal paths between BMI, reported trauma, CRP, and MDD. Each arrow represents the direction of MR analyses pointing from the exposure to the outcome. Arrow thickness is designed to approximate the strength of each effect. The dashed arrow represents a significant finding from generalized summary data–based MR, which was not confirmed by our sensitivity analyses. Gray arrows represent a nonsignificant path after multiple testing correction. BMI, body mass index; CRP, C-reactive protein; GSMR, generalized summary data–based MR; MDD, major depressive disorder; MR, Mendelian randomization.

Our study suggests both a phenotypic association and a putative causal MR effect of BMI on reported trauma, MDD, and CRP. BMI has been phenotypically and causally associated with CRP in previous studies (59,60), and our MR results are consistent with a causal role such that a 1-kg/m^2^ increase in BMI can result in a 0.37-mg/L increase in CRP. In addition, we found results consistent with a 1-kg/m^2^ increase in BMI resulting in 16% higher odds of reporting a stressful childhood life event as traumatic and 32% higher odds of developing MDD. Finally, we also show that MDD is associated with a 0.17 kg/m^2^ higher BMI. We should note that the effect of BMI on childhood trauma and of MDD on BMI did not survive our sensitivity analyses and should therefore be investigated further. In addition, our MR-CAUSE analyses indicate that the effect of BMI on MDD is best explained by a shared model, which suggests that another process may influence both traits, whereas the effect of MDD on BMI is best described by a causal model. BMI has previously been associated with many traits (61) including MDD (62, 63, 64) and lifetime trauma (65, 66, 67), but we sought to investigate these traits in tandem and to illuminate their potential causative relationships (see Figure 4 for a full schematic). We note that once BMI is controlled for, only the bidirectional association between MDD and reported trauma remains, suggesting a causal relationship that is independent of BMI. Although previous studies have shown genetic associations between MDD and increased or decreased appetite and weight (68), as well as between MDD and increased weight gain and hypersomnia (69), to our knowledge, this is the first study to investigate the bidirectional association between BMI, MDD, CRP, and childhood trauma, indicating that once BMI is controlled for, only the association between MDD and childhood trauma remains. We should also note that the relationship between BMI and MDD is difficult to disentangle, with some studies indicating that depression may increase the genetic susceptibility to higher BMI (64,70) and others, including ours, suggesting that there is a high degree of pleiotropy between these traits, making it hard to disentangle the directionality of the relationship (24). The longitudinal association of CRP with MDD also cannot be ruled out, with several studies suggesting such a relationship (71, 72, 73).

Our study has several limitations. First, although the UK Biobank remains an immensely valuable resource for science and research, it contains healthy volunteer selection bias and is therefore unrepresentative of a true control population or a clinically diagnosed psychiatric population (74). Second, studies have suggested that extreme levels of CRP are associated with treatment-resistant depression, and although there is an indication of large CRP concentrations in the UK Biobank sample, the depression phenotype is derived from a self-report. Therefore, a sample capturing both extreme CRP concentrations and a severe depression phenotype may provide further clarification (75). Third, we are aware that even in large, ascertained cohorts such as the UK Biobank, there are confounding factors that we are not able to control for (74) that may influence our phenotypic analyses. For example, childhood trauma has been associated with poor sleep quality and detrimental physical health outcomes (76). However, the joint use of MR on the same phenotypes should help overcome this limitation. Fourth, CRP is often considered as a downstream target of inflammation, which itself encompasses many inflammatory markers (77). Therefore, we may not be capturing discrete inflammatory effects but rather a more global state of inflammation, which may not apply to all MDD diagnoses. Fifth, although we used the most powerful GWASs available for MR analyses, it is possible that the effects of some single nucleotide polymorphisms are missed owing to differences in phenotyping and that our results are biased due to weak genetic instruments, especially in the case of the MDD and reported trauma phenotypes, as shown in our sensitivity analyses by the *I*^2^ statistic. Finally, although our findings suggest that BMI may be a major confounding factor when investigating the CRP-MDD association, other studies do suggest that increased CRP in patients with MDD compared with control subjects cannot be fully explained by BMI (78). Thus, more research with deeper phenotyping of MDD may help better explain this association.

### Conclusions

In conclusion, this study replicates previously reported phenotypic associations using UK Biobank data (13) and demonstrates that BMI and trauma are associated with MDD and CRP. This study also highlights the effect that BMI can have on MDD, CRP, and reported trauma using a causal MR framework, confirming its role as a strong confounding factor. Finally, this study highlights the bidirectional causal relationship between MDD and childhood reported trauma, which may explain the frequent co-occurrence of these two phenotypes. Future studies focusing on different depression subtypes, abnormally high CRP levels, and a larger number of inflammatory markers may further our knowledge of these complex interactions.

## Supplementary Material

Supplementary Material

Key Resources Table
