# Supplementary material for: Assessing the Evidence for Causal Associations Between Body Mass Index, C-Reactive Protein, Depression, and Reported Trauma Using Mendelian Randomization
Source: Biol Psychiatry Glob Open Sci. 2022 Jan 28;3(1):110–8. doi: 10.1016/j.bpsgos.2022.01.003 (PMC9874165; doi:10.1016/j.bpsgos.2022.01.003)
Supplement: Supplementary Material [file mmc1.pdf]

Assessing the Evidence for Causal Associations Between Body Mass Index, C-Reactive Protein, Depression and Reported Trauma Using Mendelian Randomization

Supplement

Table of Contents

1. Phenotype definitions from the UK Biobank 2

2. Samples used for Mendelian randomization 4

3. Outline of the HEIDI outlier method used in GSMR 5

4. Output from phenotypic analyses - associations with CRP 6

5. Output from phenotypic analyses - associations with MDD 7

6. All GSMR analyses in table form 8

7. Figures from all GSMR analyses 9

8. Sensitivity analyses using other MR methods 21

9. mtCOJO sensitivity analyses results 33

10. cis-CRP SNP effects on MDD and childhood trauma 35

11. Multivariable MR analyses of all traits on MDD 36

12. Testing the strength of our genetic instruments 37

13. Testing for shared or causal effects between CRP, reported trauma, BMI and MDD using MR-CAUSE 38

14. Supplementary References 40

1. Phenotype definitions from the UK Biobank

a. Major depressive disorder phenotyping

Models on the MDD phenotype included individuals with probable lifetime MDD based on responses to questions in the World Health Organization World Mental Health Composite International Diagnostic Interview (WHO WMH-CIDI)(1)which were incorporated into the UK Biobank mental health questionnaire. Cases were defined as anyone who scored "Yes" for any of the three MDD questions ("Probable recurrent major depression (severe)", "Probably recurrent major depression (moderate)" and "Single probably major depression episode"), collapsing them into a final binary "No MDD/Yes MDD" variable for the analyses, in line with a previous study design, which we are attempting to replicate (2, 3). Note, cases were excluded if they also self-reported a diagnosis of bipolar disorder. Controls were defined as anyone who fell into the "No MDD" category. See Table 1 for a full breakdown of MDD cases in our sample.

b. Inflammatory marker quantification

Circulating CRP concentrations were measured in serum using an immunoturbidimetric high sensitivity analysis methodology (AU5400, Beckhman Coulter) and were available for each participant. CRP quantities (pg/mL) were log-transformed to achieve a normal distribution.

c. Genotyping and polygenic scores (PGS)

Genetic data came from a full release of the UK Biobank data (4). Genotype data from two overlapping arrays underwent quality control, imputation, and were limited to common variants (minor allele frequency > 0.01), which were either directly genotyped or imputed with high confidence (5, 6). Individuals who were related (KING r > 0.044) were removed using a greedy algorithm designed to retain as many individuals as possible, as were individuals with discordant data (X-chromosome homozygosity < 0.9 for males and > 0.5 for females).

Polygenic risk scores for MDD and BMI were calculated on the UK Biobank sample using PRSice v2.(7) Imputed variants for calculating PRS were limited to common variants (MAF > 0.01) with a call rate of > 98%, that were in approximate Hardy-Weinberg equilibrium (HWE test $P > 10^{-8}$). P-value thresholds for the inclusion of SNPs in the genetic scores for the analyses were based on those found to optimise predictive accuracy in the original publications of the datasets (MDD < 0.05, BMI < 0.2) (8, 9).

d. Trauma phenotyping

The trauma phenotype used in this study was based upon previous research, which this study was aiming to replicate (2). The trauma items taken from the UK Biobank were as follows:

Childhood trauma, consisting of six categories (“Prefer not to say” (-1), “Never true” (0), “Rarely true” (1) “Sometimes true” (2), “Often” (3), “Very often true” (4)):

- Felt loved as a child
- Physically abused by family as a child
- Felt hated by family member as a child
- Sexually molested as a child
- Someone to take to doctor when needed as a child

Adulthood trauma, consisting of six categories (“Prefer not to say” (-1), “Never true” (0), “Rarely true” (1) “Sometimes true” (2), “Often” (3), “Very often true” (4)):

- Physical violence by partner or ex-partner as an adult
- Belittlement by partner or ex-partner as an adult
- Sexual interference by partner or ex-partner without consent as an adult

Physical trauma, consisting of four categories (“Prefer not to say” (-1), “Never” (0), “Yes, but not in the past 12 months” (1), “Yes, within the past 12 months” (2)):

- Victim of physically violent crime
- Been in serious accident believed to be life-threatening

A mean score across all five questions was calculated for each individual, omitting “Prefer not to say”. The mean score was then collapsed into binary “No Trauma” and “Yes Trauma” scores, with “Yes Trauma” categorizing anyone who had a mean score of 0.5 and above and “No Trauma” categorizing anyone who had a mean score of less than 0.

2. Samples used for Mendelian randomization

a. MDD GWAS

For all analyses we used the latest and largest MDD GWAS from the Psychiatric Genomic Consortium. This GWA meta-analysis consisted of 135,458 cases and 344,901 controls of European ancestry (8). In total, this GWAS reported 44 significant genetic loci and could explain 8.7% of heritability in a lifetime depression diagnosis.

b. CRP GWAS

For all analyses we used the largest CRP GWAS from the CHARGE Inflammation Working Group, consisting of 204,402 individuals of European ancestry (10). This GWAS reported 58 significant genetic loci that could explain up to 7% of the variance in circulating CRP levels.

c. BMI GWAS

For all analyses, we used a meta-analysis of the largest BMI GWAS combining the UK Biobank and the GIANT consortia(9). This sample consisted of 694,649 individuals of European ancestry and identified 463 signals in 346 loci, and could explain 17.4% of heritability in BMI.

d. Childhood Trauma GWAS

For all analyses we used the latest and largest childhood trauma GWAS (11). The sample consisted of 185,414 individuals of European ancestry. This GWAS reported 14 genome-wide significant loci, and could explain 10% of heritability in childhood trauma.

3. Outline of the HEIDI outlier method used in GSMR

The basic idea of the HEIDI outlier test is to test where there is a significant difference between b^xy estimated at an instrument i (i.e., b^xy(i)) and b^xy estimated at a target SNP that shows a strong association with the exposure. It performs a calculation to identify pleiotropic SNPs that have an effect on both the exposure and the outcome, taking into account the LD correlation between the two SNPs (estimated from a reference sample with individual-level genotypes) and tests the deviation of each SNP from the causal model using the $\chi 2$-statistic and removes the SNPs with P-values < 0.01

4. Output from phenotypic analyses - associations with CRP

Table S1. Output from linear models with CRP as the outcome of interest

	Model 1	Model 2	Model 3	Model 4	Model 5	Model 6
Childhood Trauma	0.02 **					
	[0.01, 0.03]					
Adulthood Trauma		0.03 ***				
		[0.02, 0.04]				
Physical Trauma			0.02			
			[-0.01, 0.04]			
MDD				0.00		
				[-0.02, 0.02]		
MDD PRS					0.00	
					[-0.01, 0.01]	
BMI PRS						0.07 ***
						[0.06, 0.08]
Smoking Status	0.05 ***	0.05 ***	0.05 ***	0.04 ***	0.04 ***	0.07 ***
	[0.04, 0.05]	[0.04, 0.05]	[0.04, 0.05]	[0.04, 0.05]	[0.04, 0.05]	[0.06, 0.08]
Age	0.09 ***	0.09 ***	0.09 ***	0.09 ***	0.09 ***	0.11 ***
	[0.08, 0.09]	[0.09, 0.10]	[0.08, 0.09]	[0.08, 0.10]	[0.08, 0.10]	[0.10, 0.12]
BMI	0.44 ***	0.44 ***	0.44 ***	0.44 ***	0.43 ***	
	[0.43, 0.44]	[0.43, 0.44]	[0.43, 0.44]	[0.43, 0.45]	[0.43, 0.44]	
Sex	-0.17 ***	-0.17 ***	-0.17 ***	-0.20 ***	-0.20 ***	-0.09 ***
	[-0.18, -0.16]	[-0.18, -0.16]	[-0.18, -0.16]	[-0.22, -0.18]	[-0.22, -0.18]	[-0.11, -0.07]
N	113481	113481	113481	30313	29870	29870
R2	0.23	0.23	0.23	0.23	0.23	0.04

All continuous predictors are mean-centered and scaled by 1 standard deviation. *** p < 0.001; ** p < 0.01; * p < 0.05.

Note: Each predictor was independently tested using a linear model. Each model was controlled for age, sex, six genomic principal components, 21 assessment centre covariates, 105 batch covariates, fasting time, smoking status and BMI (with the exception of when BMI was the predictor of interest). This table omits six genomic principal components, 21 assessment centre covariates and 105 batch covariates for ease of visualisation. Values displayed signify the beta and a 95% confidence interval. Significance is determined by the asterisk key at the bottom of the table. MDD = major depressive disorder; PRS = polygenic risk score; BMI = body mass index; N = number.

5. Output from phenotypic analyses - associations with MDD

Table S2. Output from logistic models with MDD as the outcome of interest

	Model 1	Model 2	Model 3	Model 4	Model 5	Model 6
Childhood Trauma		0.69 ***				
		[0.63, 0.75]				
Adulthood Trauma			0.90 ***			
			[0.84, 0.97]			
Physical Trauma				0.51 ***		
				[0.38, 0.64]		
BMI PRS						464.90
						[-120.96, 1050.76]
CRP	0.01					
	[-0.02, 0.04]					
Smoking Status	0.31 ***	0.27 ***	0.26 ***	0.30 ***	0.31 ***	0.32 ***
	[0.27, 0.35]	[0.23, 0.31]	[0.21, 0.30]	[0.26, 0.34]	[0.27, 0.35]	[0.28, 0.36]
Age	-0.03 ***	-0.03 ***	-0.02 ***	-0.03 ***	-0.03 ***	-0.03 ***
	[-0.03, -0.02]	[-0.03, -0.02]	[-0.03, -0.02]	[-0.03, -0.02]	[-0.03, -0.02]	[-0.03, -0.02]
BMI	0.95 ***	0.86 ***	0.92 ***	0.96 ***	0.97 ***	
	[0.77, 1.13]	[0.70, 1.03]	[0.76, 1.09]	[0.80, 1.12]	[0.81, 1.13]	
Sex	-0.69 ***	-0.67 ***	-0.55 ***	-0.71 ***	-0.69 ***	-0.64 ***
	[-0.74, -0.63]	[-0.72, -0.61]	[-0.61, -0.50]	[-0.76, -0.65]	[-0.74, -0.63]	[-0.70, -0.59]
N	30137	30137	30137	30137	30137	30137
AIC	34479.59	33954.89	33722.89	34424.07	34477.76	34613.15
BIC	34604.29	34079.59	33847.59	34548.77	34594.15	34729.54
Pseudo R2	0.05	0.08	0.09	0.06	0.05	0.05

*** p < 0.001; ** p < 0.01; * p < 0.05.

Note: Each predictor was independently tested using a logistic model. Each model was controlled for age, sex, six genomic principal components, 21 assessment centre covariates, 105 batch covariates, fasting time, smoking status and BMI (with the exception of when BMI was the predictor of interest). This table omits six genomic principal components, 21 assessment centre covariates and 105 batch covariates for ease of visualisation. Values displayed signify the beta and a 95% confidence interval. Significance is determined by the asterisk key at the bottom of the table. MDD = major depressive disorder; PRS = polygenic risk score; BMI = body mass index; N = number.

6. All GSMR analyses in table form

Table S3. GSMR output table

Exposure	Outcome	Beta xy	Lower CI	Upper CI	P	N SNPs
BMI	CRP	0.3676	0.3487	0.3864	>0.0000	1175
CRP	BMI	-0.0016	-0.0108	0.0076	0.7325	55
BMI	MDD	0.1532	0.1308	0.1756	>0.0000	1111
MDD	BMI	0.1664	0.1406	0.1921	>0.0000	24
BMI	Childhood trauma	0.0882	0.0729	0.1035	>0.0000	1184
Childhood trauma	BMI	0.1006	0.0480	0.1532	0.0002	10
CRP	MDD	0.0201	-0.0034	0.0436	0.0930	88
MDD	CRP	0.0596	0.0169	0.1023	0.0062	43
MDD	Childhood trauma	0.1843	0.1480	0.2206	>0.0000	42
Childhood trauma	MDD	0.5108	0.3595	0.6622	>0.0000	11
CRP	Childhood trauma	0.0117	-0.0008	0.0241	0.0658	130
Childhood trauma	CRP	0.1031	-0.0165	0.2227	0.0912	11

Note: Each row represents one Mendelian randomization test between the exposure and the outcome. MDD = major depressive disorder; BMI = body mass index; Beta xy = beta of the exposure (x) on the outcome (y); Lower CI = lower 95% confidence interval; Upper CI = upper 95% confidence interval; P = p-value; nSNP = number of SNPs used in the analyses.

7. Figures from all GSMR analyses

a. MDD on CRP

43 index SNPs were obtained from the clumping analysis with $p < 5.0e-08$ and LD $r2 < 0.05$. 43 index SNPs were retained after HEIDI-outlier analysis.

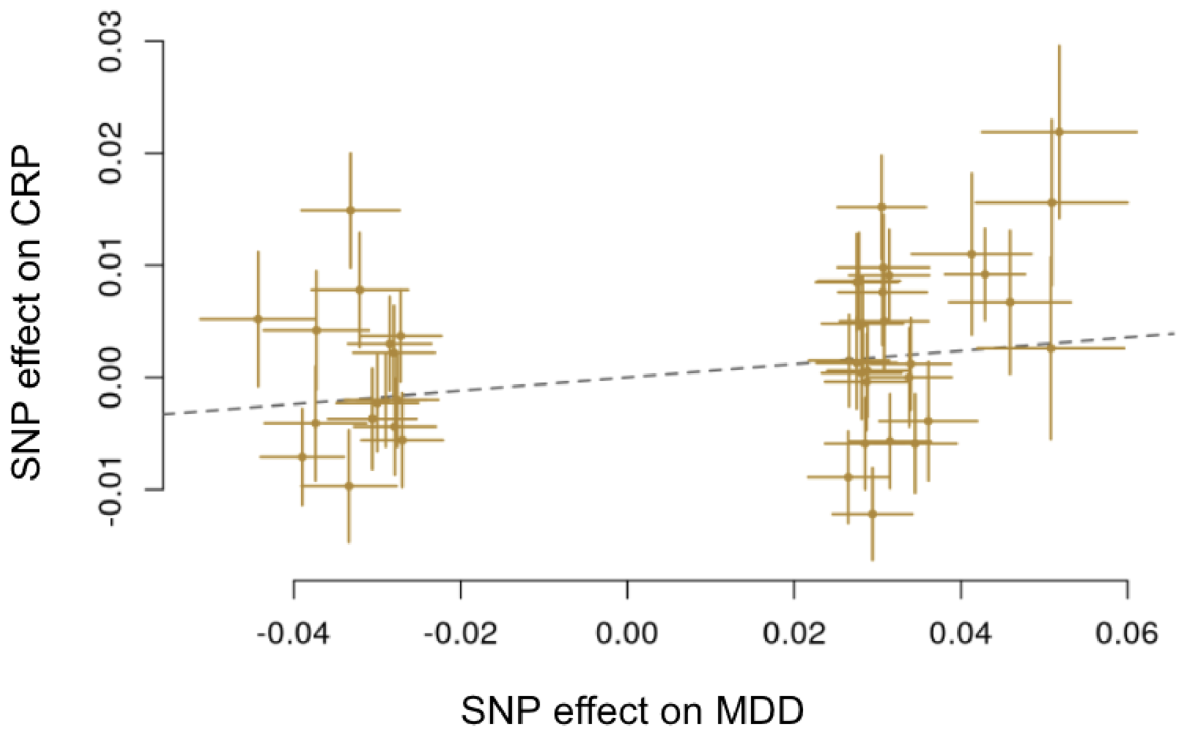

Figure S1. GSMR chart with MDD as exposure and CRP as the outcome

This figure illustrates the MR relationship between MDD and CRP. SNP effects associated with MDD are displayed on the x-axis and SNP effects associated with CRP are displayed on the y-axis. Each point represents the beta coefficient of these two traits. The horizontal line on each point represents the standard error associated with the exposure and the vertical line on each point represents the standard error associated with the outcome. The dotted line represents the regression line between these two traits.

b. CRP on MDD

94 index SNPs were obtained from the clumping analysis with p < 5.0e-08 and LD r2 < 0.05. 6 pleiotropic SNPs were filtered by HEIDI-outlier analysis. 88 index SNPs were retained after HEIDI-outlier analysis.

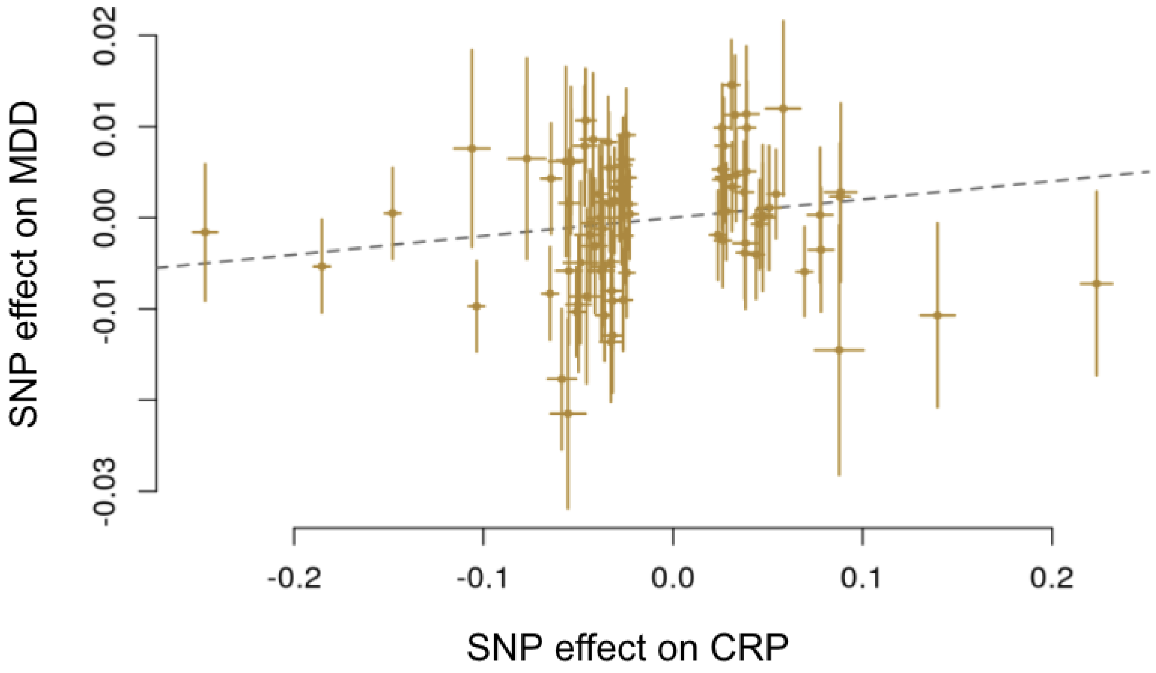

Figure S2. GSMR chart with CRP as exposure and MDD as the outcome

This figure illustrates the MR relationship between CRP and MDD. SNP effects associated with CRP are displayed on the x-axis and SNP effects associated with MDD are displayed on the y-axis. Each point represents the beta coefficient of these two traits. The horizontal line on each point represents the standard error associated with the exposure and the vertical line on each point represents the standard error associated with the outcome. The dotted line represents the regression line between these two traits.

c. BMI on CRP

1224 index SNPs were obtained from the clumping analysis with $p < 5.0e-08$ and LD $r2 < 0.05$. 49 pleiotropic SNPs were filtered by HEIDI-outlier analysis. 1175 index SNPs were retained after HEIDI-outlier analysis.

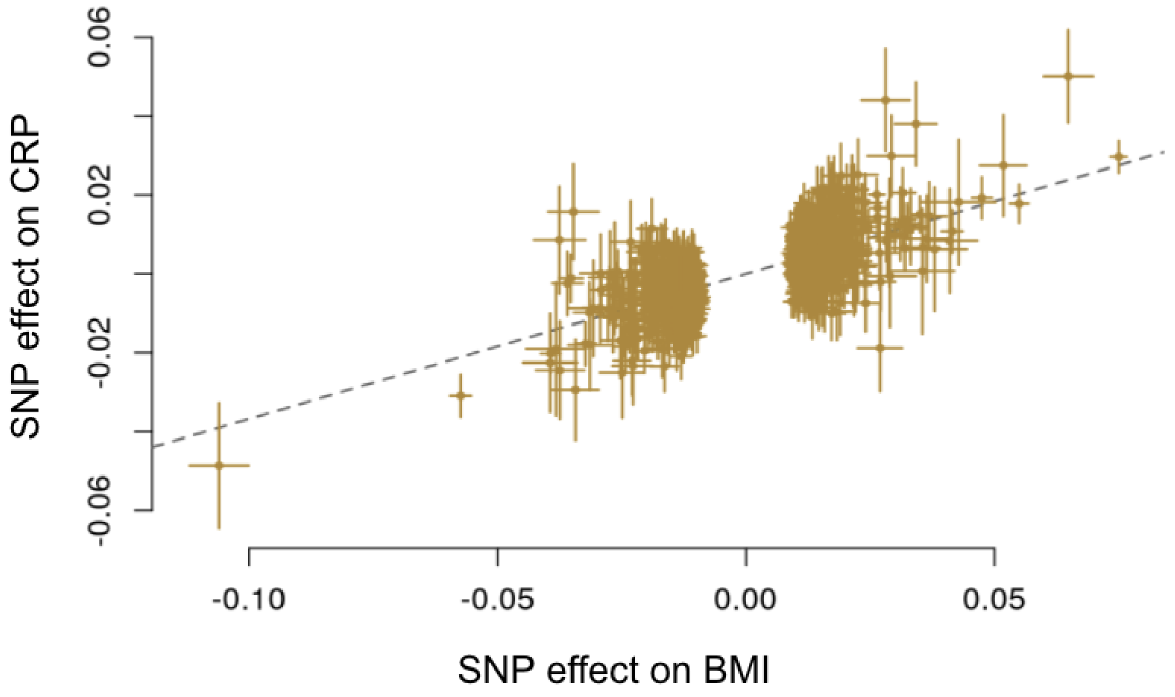

Figure S3. GSMR chart with BMI as exposure and CRP as the outcome

This figure illustrates the MR relationship between BMI and CRP. SNP effects associated with BMI are displayed on the x-axis and SNP effects associated with CRP are displayed on the y-axis. Each point represents the beta coefficient of these two traits. The horizontal line on each point represents the standard error associated with the exposure and the vertical line on each point represents the standard error associated with the outcome. The dotted line represents the regression line between these two traits.

d. CRP on BMI

88 index SNPs are obtained from the clumping analysis with p < 5.0e-08 and LD r2 < 0.05. 33 pleiotropic SNPs are filtered by HEIDI-outlier analysis. 55 index SNPs are retained after HEIDI-outlier analysis.

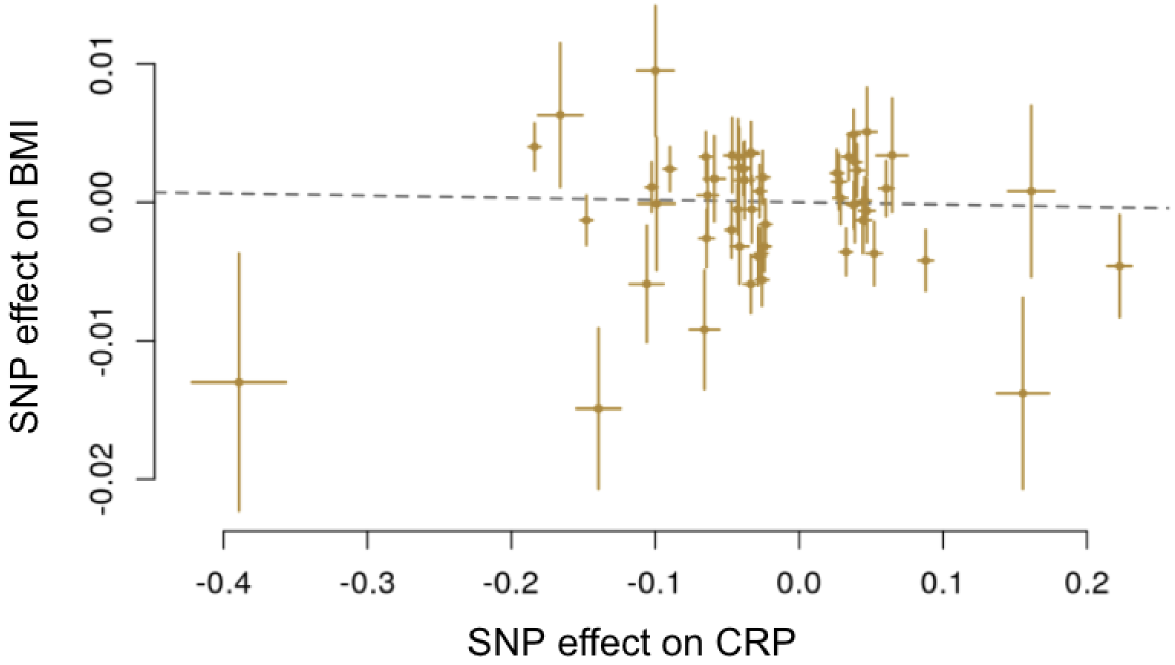

Figure S4. GSMR chart with CRP as exposure and BMI as the outcome

This figure illustrates the MR relationship between CRP and BMI. SNP effects associated with CRP are displayed on the x-axis and SNP effects associated with BMI are displayed on the y-axis. Each point represents the beta coefficient of these two traits. The horizontal line on each point represents the standard error associated with the exposure and the vertical line on each point represents the standard error associated with the outcome. The dotted line represents the regression line between these two traits.

e. BMI on MDD

1199 index SNPs are obtained from the clumping analysis with $p < 5.0e-08$ and LD $r2 < 0.05$. 88 pleiotropic SNPs are filtered by HEIDI-outlier analysis. 1111 index SNPs are retained after HEIDI-outlier analysis.

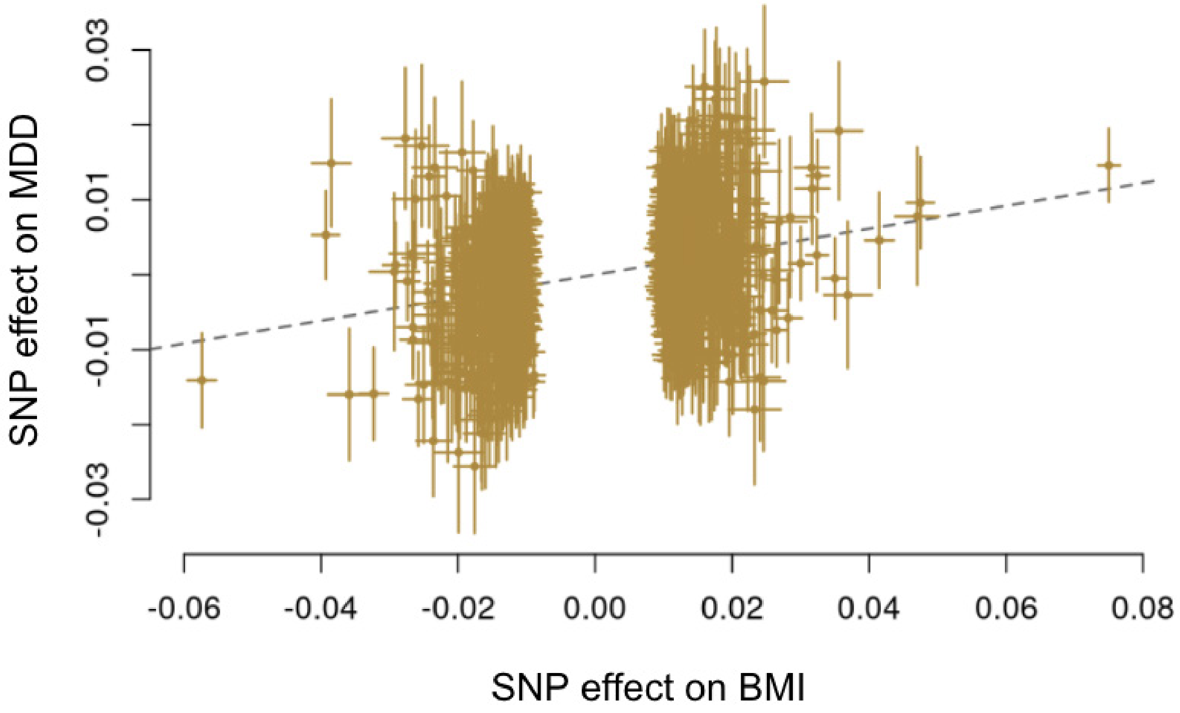

Figure S5. GSMR chart with BMI as exposure and MDD as the outcome

This figure illustrates the MR relationship between BMI and MDD. SNP effects associated with BMI are displayed on the x-axis and SNP effects associated with MDD are displayed on the y-axis. Each point represents the beta coefficient of these two traits. The horizontal line on each point represents the standard error associated with the exposure and the vertical line on each point represents the standard error associated with the outcome. The dotted line represents the regression line between these two traits.

f. MDD on BMI

35 index SNPs are obtained from the clumping analysis with p < 5.0e-08 and LD r2 < 0.05. 11 pleiotropic SNPs are filtered by HEIDI-outlier analysis. 24 index SNPs are retained after HEIDI-outlier analysis.

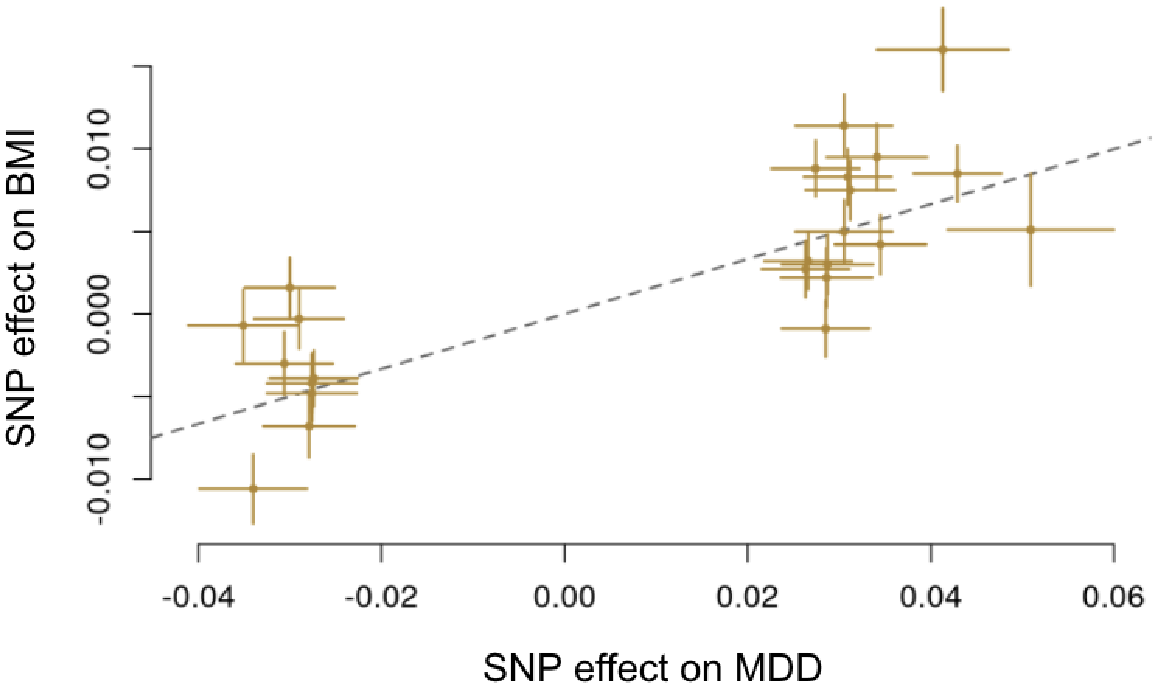

Figure S6. GSMR chart with MDD as exposure and BMI as the outcome

This figure illustrates the MR relationship between MDD and BMI. SNP effects associated with MDD are displayed on the x-axis and SNP effects associated with BMI are displayed on the y-axis. Each point represents the beta coefficient of these two traits. The horizontal line on each point represents the standard error associated with the exposure and the vertical line on each point represents the standard error associated with the outcome. The dotted line represents the regression line between these two traits.

g. Childhood trauma on CRP

11 index SNPs are obtained from the clumping analysis with p < 5.0e-08 and LD r2 < 0.05. 11 index SNPs are retained after HEIDI-outlier analysis.

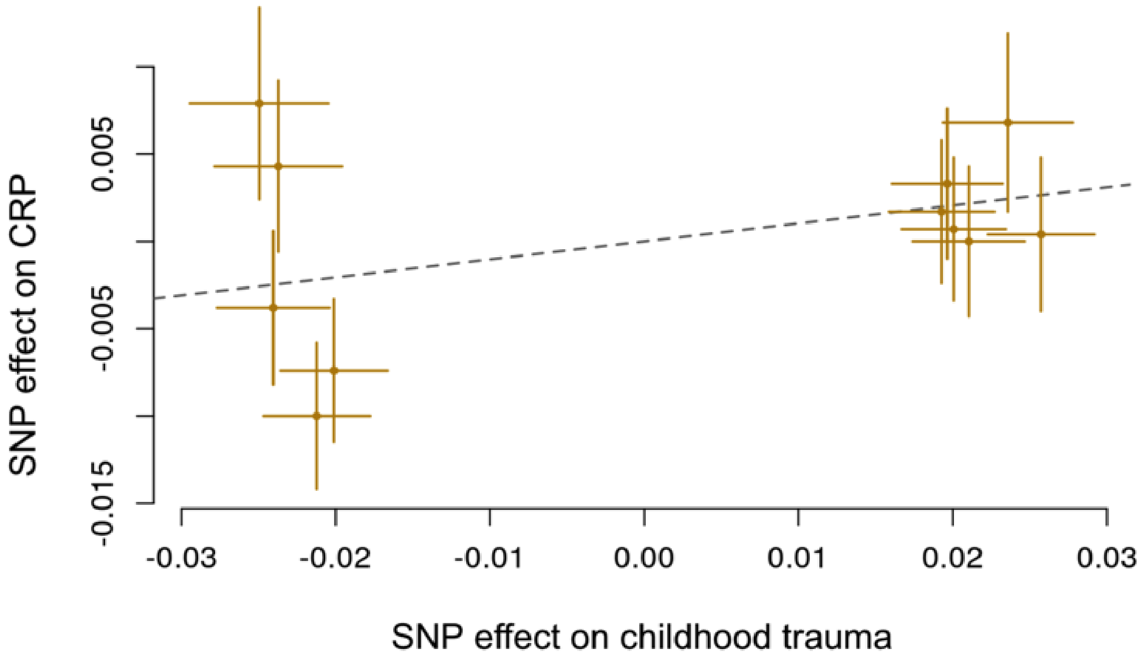

Figure S7. GSMR chart with childhood trauma as exposure and CRP as the outcome

This figure illustrates the MR relationship between childhood trauma and CRP. SNP effects associated with childhood trauma are displayed on the x-axis and SNP effects associated with CRP are displayed on the y-axis. Each point represents the beta coefficient of these two traits. The horizontal line on each point represents the standard error associated with the exposure and the vertical line on each point represents the standard error associated with the outcome. The dotted line represents the regression line between these two traits.

h. CRP on childhood trauma

133 index SNPs are obtained from the clumping analysis with p < 5.0e-08 and LD r2 < 0.05. 3 pleiotropic SNPs are filtered by HEIDI-outlier analysis. 130 index SNPs are retained after HEIDI-outlier analysis.

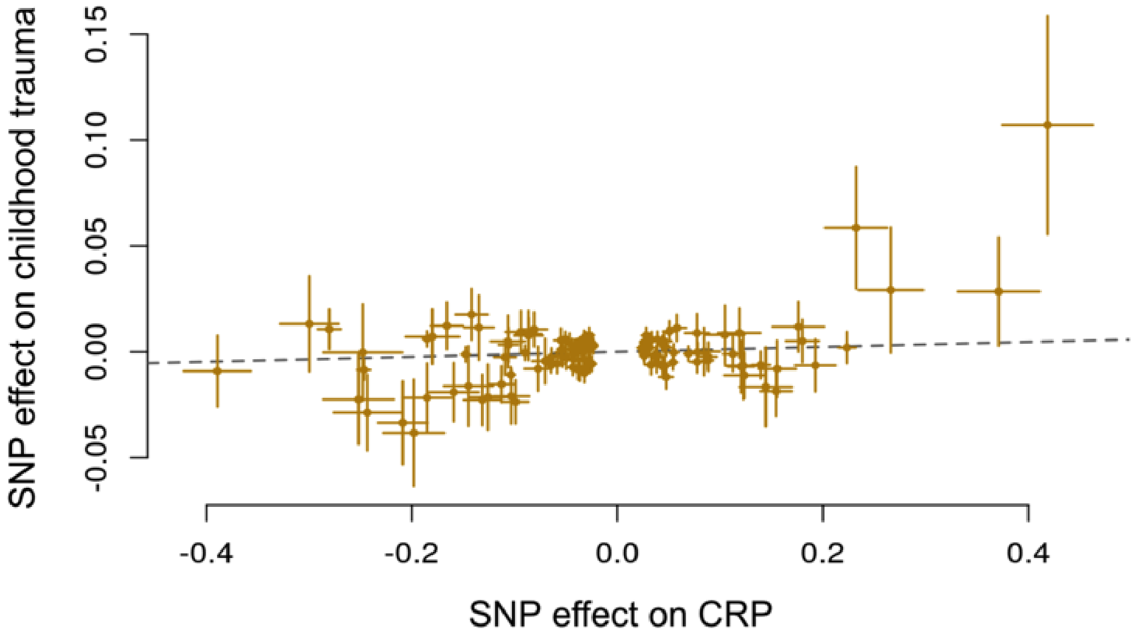

Figure S8. GSMR chart with CRP as exposure and childhood trauma as the outcome

This figure illustrates the MR relationship between CRP and childhood trauma. SNP effects associated with CRP are displayed on the x-axis and SNP effects associated with childhood trauma are displayed on the y-axis. Each point represents the beta coefficient of these two traits. The horizontal line on each point represents the standard error associated with the exposure and the vertical line on each point represents the standard error associated with the outcome. The dotted line represents the regression line between these two traits.

i. Childhood trauma on MDD

11 index SNPs are obtained from the clumping analysis with $p < 5.0e-08$ and LD $r2 < 0.05$. 11 index SNPs are retained after HEIDI-outlier analysis.

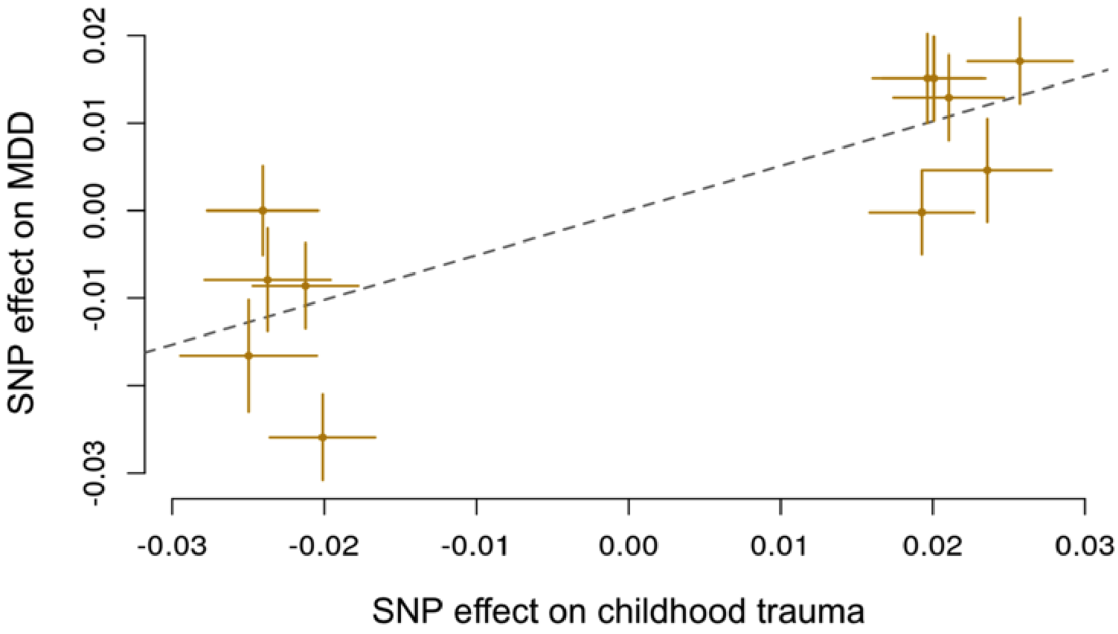

Figure S9. GSMR chart with childhood trauma as exposure and MDD as the outcome

This figure illustrates the MR relationship between childhood trauma and MDD. SNP effects associated with childhood trauma are displayed on the x-axis and SNP effects associated with MDD are displayed on the y-axis. Each point represents the beta coefficient of these two traits. The horizontal line on each point represents the standard error associated with the exposure and the vertical line on each point represents the standard error associated with the outcome. The dotted line represents the regression line between these two traits.

j. MDD on childhood trauma

43 index SNPs are obtained from the clumping analysis with p < 5.0e-08 and LD r2 < 0.05. 1 pleiotropic SNP was filtered by HEIDI-outlier analysis. 42 index SNPs are retained after HEIDI-outlier analysis.

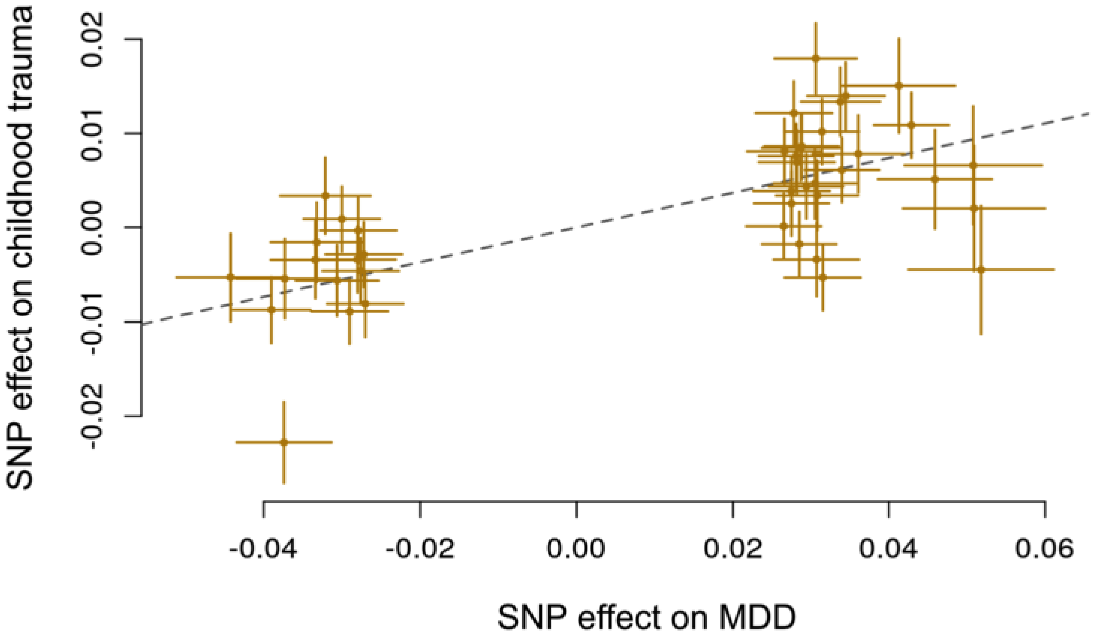

Figure S10. GSMR chart with MDD as exposure and childhood trauma as the outcome

This figure illustrates the MR relationship between MDD and childhood trauma. SNP effects associated with MDD are displayed on the x-axis and SNP effects associated with childhood trauma are displayed on the y-axis. Each point represents the beta coefficient of these two traits. The horizontal line on each point represents the standard error associated with the exposure and the vertical line on each point represents the standard error associated with the outcome. The dotted line represents the regression line between these two traits.

k. BMI on childhood trauma

1223 index SNPs are obtained from the clumping analysis with p < 5.0e-08 and LD r2 < 0.05. 39 pleiotropic SNPs are filtered by HEIDI-outlier analysis. 1184 index SNPs are retained after HEIDI-outlier analysis.

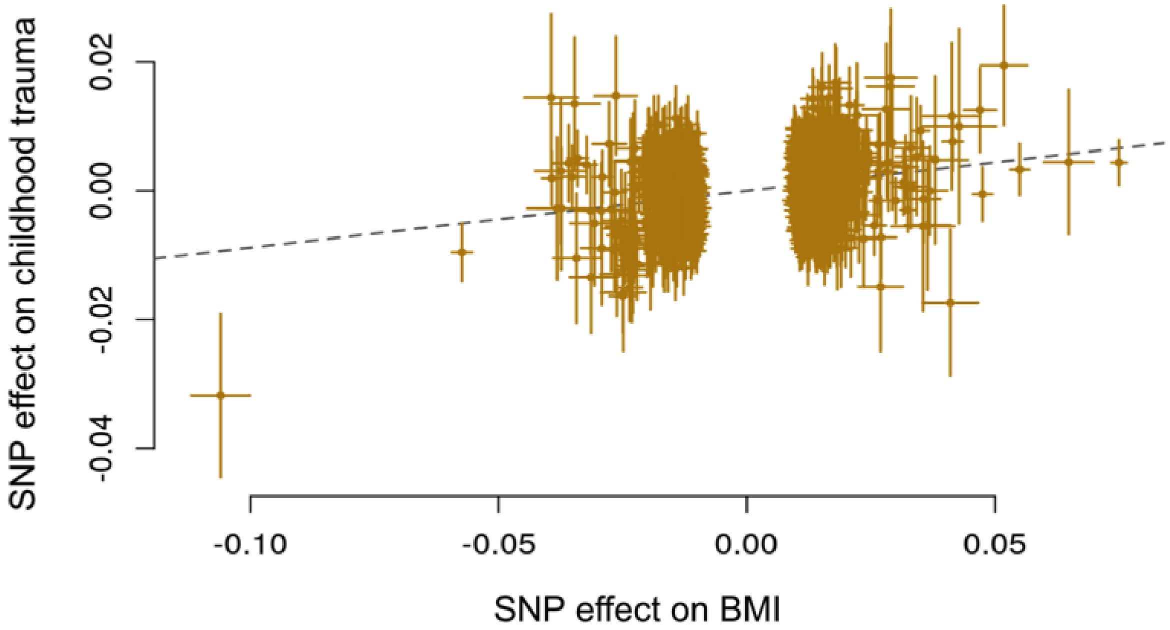

Figure S11. GSMR chart with BMI as exposure and childhood trauma as the outcome

This figure illustrates the MR relationship between BMI and childhood trauma. SNP effects associated with BMI are displayed on the x-axis and SNP effects associated with childhood trauma are displayed on the y-axis. Each point represents the beta coefficient of these two traits. The horizontal line on each point represents the standard error associated with the exposure and the vertical line on each point represents the standard error associated with the outcome. The dotted line represents the regression line between these two traits.

l. Childhood trauma on BMI

10 index SNPs are obtained from the clumping analysis with $p < 5.0e-08$ and LD $r2 < 0.05$. 10 index SNPs are retained after HEIDI-outlier analysis.

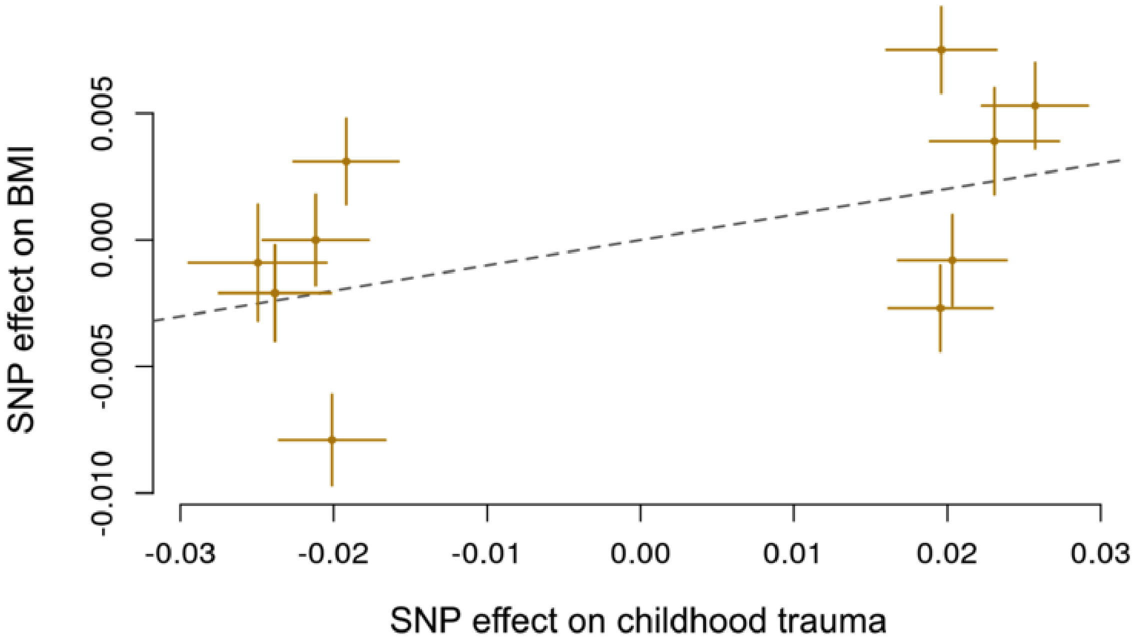

Figure S12. GSMR chart with childhood trauma as exposure and BMI as the outcome

This figure illustrates the MR relationship between childhood trauma and BMI. SNP effects associated with childhood trauma are displayed on the x-axis and SNP effects associated with BMI are displayed on the y-axis. Each point represents the beta coefficient of these two traits. The horizontal line on each point represents the standard error associated with the exposure and the vertical line on each point represents the standard error associated with the outcome. The dotted line represents the regression line between these two traits.

8. Sensitivity analyses using other MR methods

a. MDD on CRP

Table S4. Sensitivity analyses of MDD (exposure) on CRP (outcome)

exposure	outcome	method	nsnp	b	lo_ci	up_ci	pval
MDD	CRP	MR Egger	39	0.31822744	-0.06834157	0.7047965	0.11513552
MDD	CRP	Weighted median	39	0.06212780	-0.01016365	0.1344192	0.09209699
MDD	CRP	Inverse variance weighted	39	0.05143699	-0.01479167	0.1176657	0.12794674
MDD	CRP	Simple mode	39	0.06243798	-0.11697313	0.2418491	0.49930515
MDD	CRP	Weighted mode	39	0.08048370	-0.08203682	0.2430042	0.33786824

Number of SNPS = nsnp / Beta = b / Lower 95% confidence interval = lo_ci / Upper 95% confidence interval = up_ci / P-value = pval

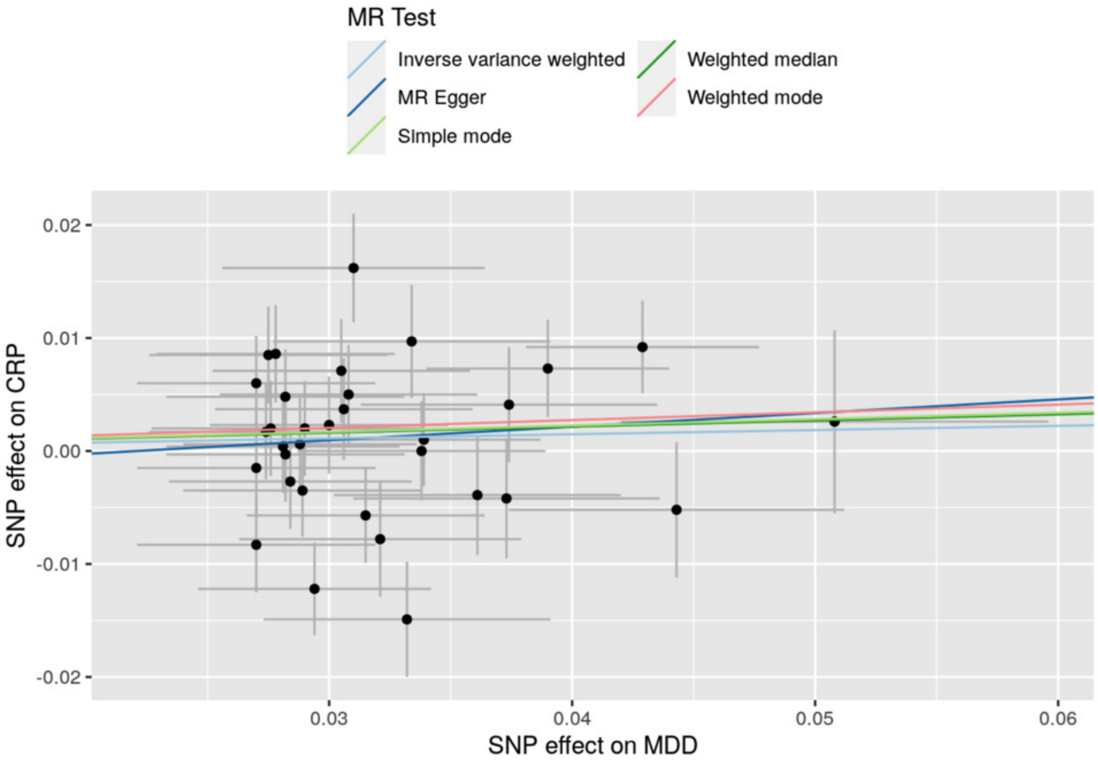

Figure S13. MR sensitivity analyses chart with MDD as exposure and CRP as the outcome

This figure illustrates the MR relationship between MDD and CRP. SNP effects associated with MDD are displayed on the x-axis and SNP effects associated with CRP are displayed on the y-axis. Each point represents the beta coefficient of these two traits. The horizontal line on each point represents the standard error associated with the exposure and the vertical line on each point represents the standard error associated with the outcome. Each coloured line represents a regression line using the MR test outlined in the legend key.

b. CRP on MDD

Table S5. Sensitivity analyses of CRP (exposure) on MDD (outcome)

exposure	outcome	method	nsnp	b	lo_ci	up_ci	pval
CRP	MDD	MR Egger	84	-0.02744912	-0.08381248	0.02891424	0.3426231
CRP	MDD	Weighted median	84	-0.01723914	-0.05750394	0.02302567	0.4013782
CRP	MDD	Inverse variance weighted	84	0.00261655	-0.02833948	0.03357258	0.8684178
CRP	MDD	Simple mode	84	-0.04855294	-0.12173694	0.02463106	0.1970851
CRP	MDD	Weighted mode	84	-0.01048489	-0.05051320	0.02954343	0.6090383

Number of SNPS = nsnp / Beta = b / Lower 95% confidence interval = lo_ci / Upper 95% confidence interval = up_ci / P-value = pval

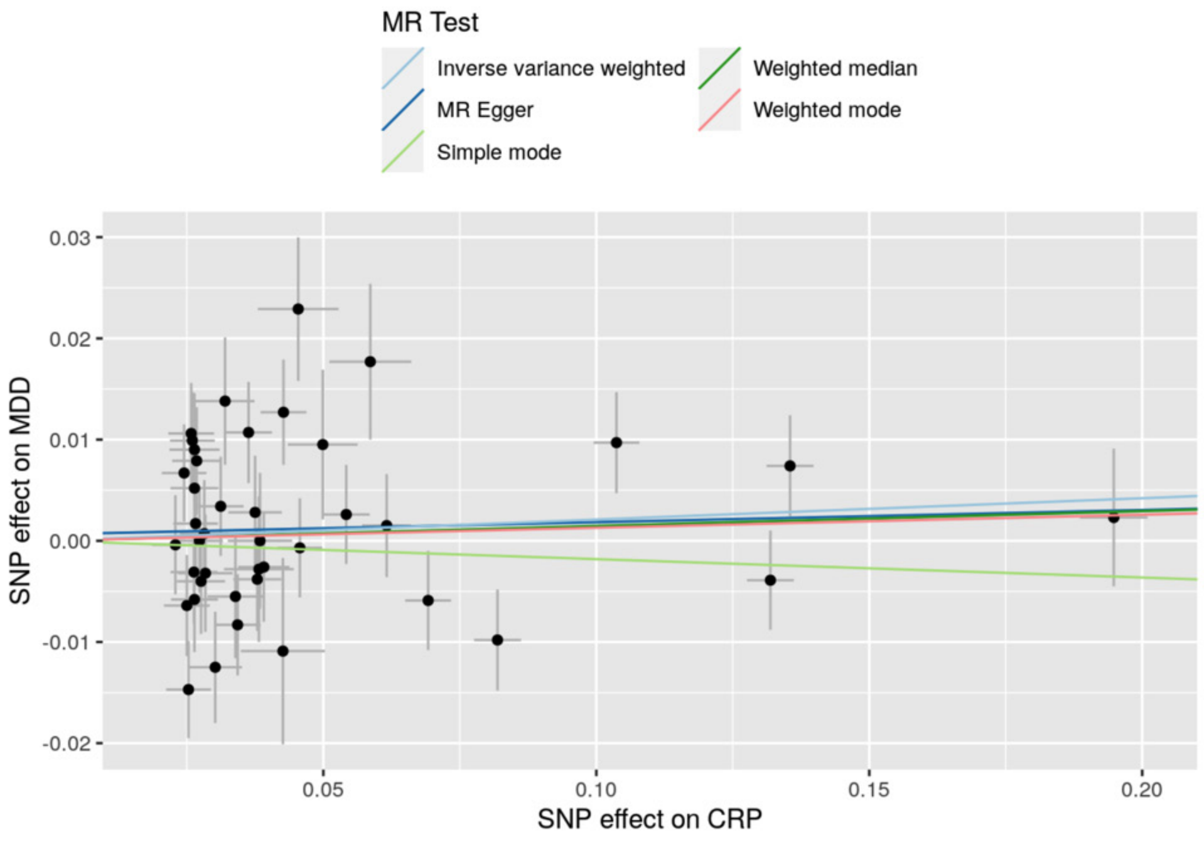

Figure S14. MR sensitivity analyses with CRP as exposure and MDD as the outcome

This figure illustrates the MR relationship between CRP and MDD. SNP effects associated with CRP are displayed on the x-axis and SNP effects associated with MDD are displayed on the y-axis. Each point represents the beta coefficient of these two traits. The horizontal line on each point represents the standard error associated with the exposure and the vertical line on each point represents the standard error associated with the outcome. Each coloured line represents a regression line using the MR test outlined in the legend key.

c. BMI on CRP

Table S6. Sensitivity analyses of BMI (exposure) on CRP (outcome)

exposure	outcome	method	nsnp	b	lo_ci	up_ci	pval
BMI	CRP	MR Egger	1207	0.4355467	0.3434297	0.5276636	8.521059e-20
BMI	CRP	Weighted median	1207	0.3587296	0.3261866	0.3912725	1.591675e-103
BMI	CRP	Inverse variance weighted	1207	0.3588448	0.3291505	0.3885391	5.040997e-124
BMI	CRP	Simple mode	1207	0.3787394	0.2538019	0.5036769	3.688743e-09
BMI	CRP	Weighted mode	1207	0.3787394	0.2900788	0.4674000	1.544415e-16

Number of SNPS = nsnp / Beta = b / Lower 95% confidence interval = lo_ci / Upper 95% confidence interval = up_ci / P-value = pval

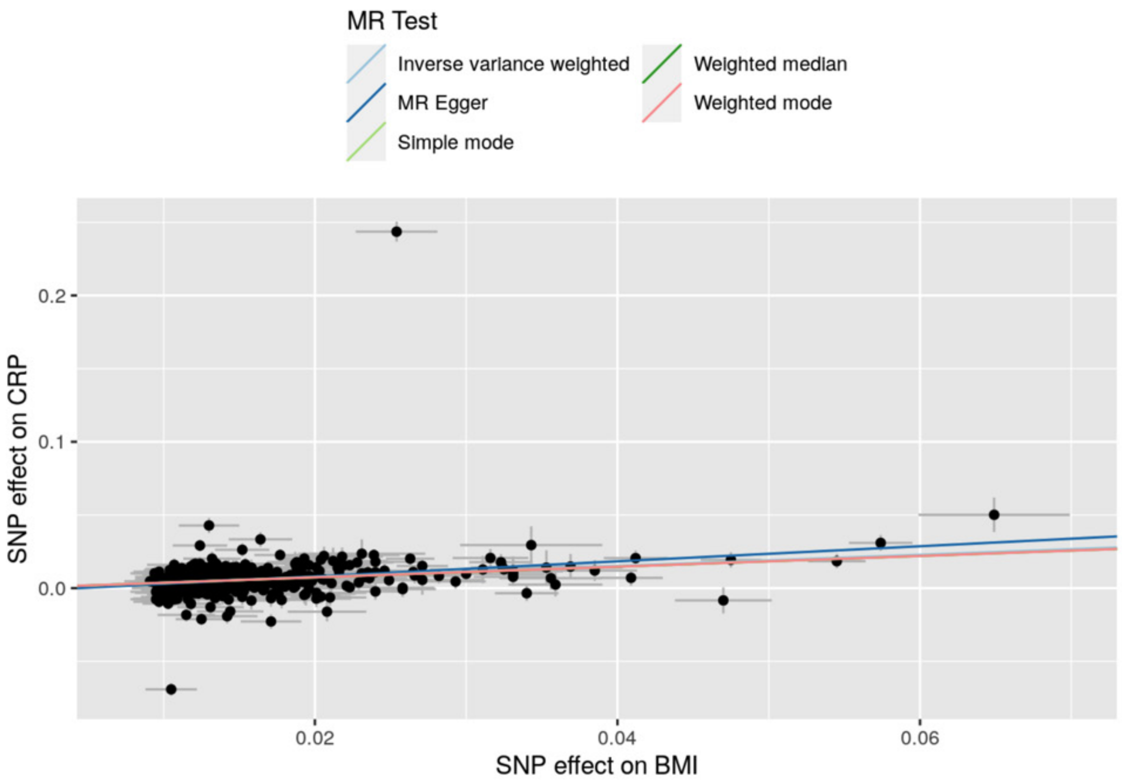

Figure S15. MR sensitivity analyses with BMI as exposure and CRP as the outcome

This figure illustrates the MR relationship between BMI and CRP. SNP effects associated with BMI are displayed on the x-axis and SNP effects associated with CRP are displayed on the y-axis. Each point represents the beta coefficient of these two traits. The horizontal line on each point represents the standard error associated with the exposure and the vertical line on each point represents the standard error associated with the outcome. Each coloured line represents a regression line using the MR test outlined in the legend key.

d. CRP on BMI

Table S7. Sensitivity analyses of CRP (exposure) on BMI (outcome)

exposure	outcome	method	nsnp	b	lo_ci	up_ci	pval
CRP	BMI	MR Egger	36	0.01672391	-0.050116781	0.08356461	0.626998883
CRP	BMI	Weighted median	36	0.03757493	0.005704457	0.06944541	0.020842910
CRP	BMI	Inverse variance weighted	36	0.06574389	0.024611739	0.10687604	0.001731585
CRP	BMI	Simple mode	36	0.02943334	-0.016234530	0.07510121	0.214854490
CRP	BMI	Weighted mode	36	0.02943334	-0.028976265	0.08784294	0.330093944

Number of SNPS = nsnp / Beta = b / Lower 95% confidence interval = lo_ci / Upper 95% confidence interval = up_ci / P-value = pval

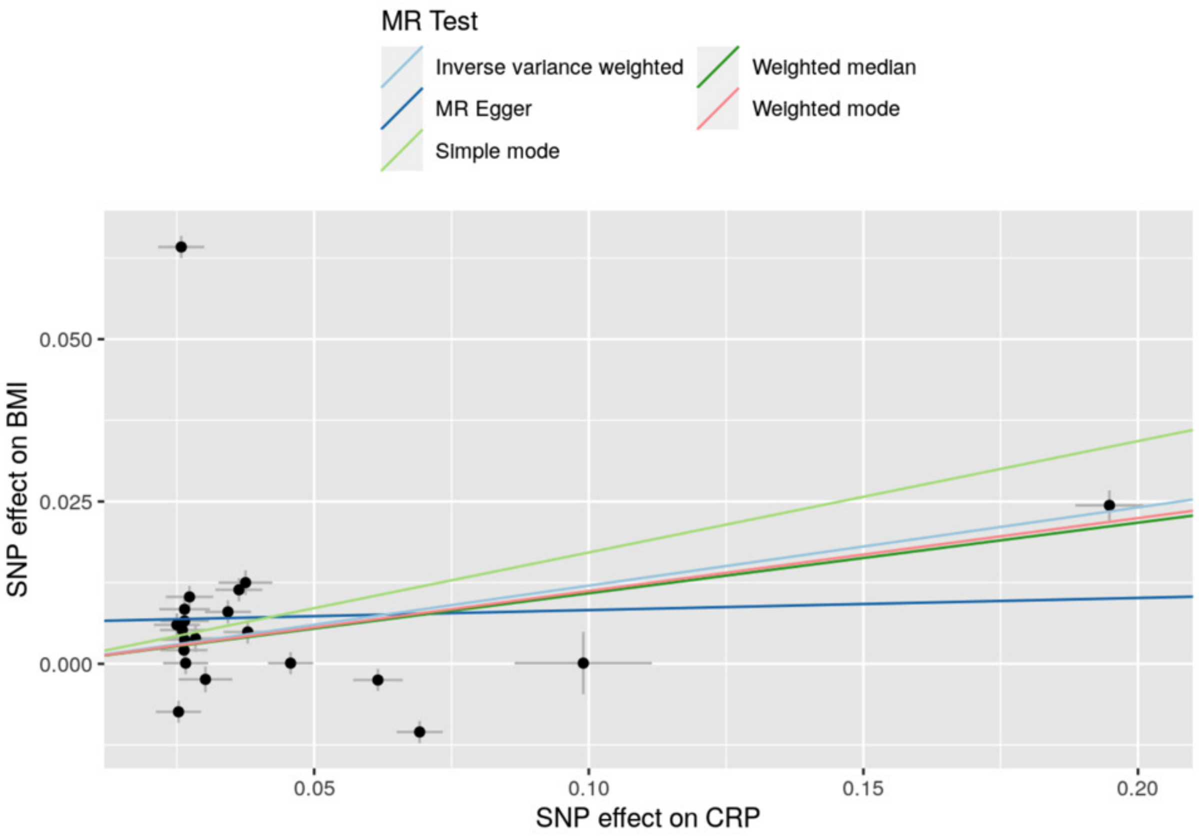

Figure S16. MR sensitivity analyses with CRP as exposure and BMI as the outcome

This figure illustrates the MR relationship between CRP and BMI. SNP effects associated with CRP are displayed on the x-axis and SNP effects associated with BMI are displayed on the y-axis. Each point represents the beta coefficient of these two traits. The horizontal line on each point represents the standard error associated with the exposure and the vertical line on each point represents the standard error associated with the outcome. Each coloured line represents a regression line using the MR test outlined in the legend key.

e. BMI on MDD

Table S8. Sensitivity analyses of BMI (exposure) on MDD (outcome)

exposure	outcome	method	nsnp	b	lo_ci	up_ci	pval
BMI	MDD	MR Egger	1178	0.05198342	-0.057009727	0.1609766	3.500799e-01
BMI	MDD	Weighted median	1178	0.11348985	0.075417559	0.1515622	5.140108e-09
BMI	MDD	Inverse variance weighted	1178	0.13039944	0.096592982	0.1642059	4.025125e-14
BMI	MDD	Simple mode	1178	0.17476487	-0.012877468	0.3624072	6.818047e-02
BMI	MDD	Weighted mode	1178	0.14926150	-0.008059769	0.3065828	6.319338e-02

Number of SNPS = nsnp / Beta = b / Lower 95% confidence interval = lo_ci / Upper 95% confidence interval = up_ci / P-value = pval

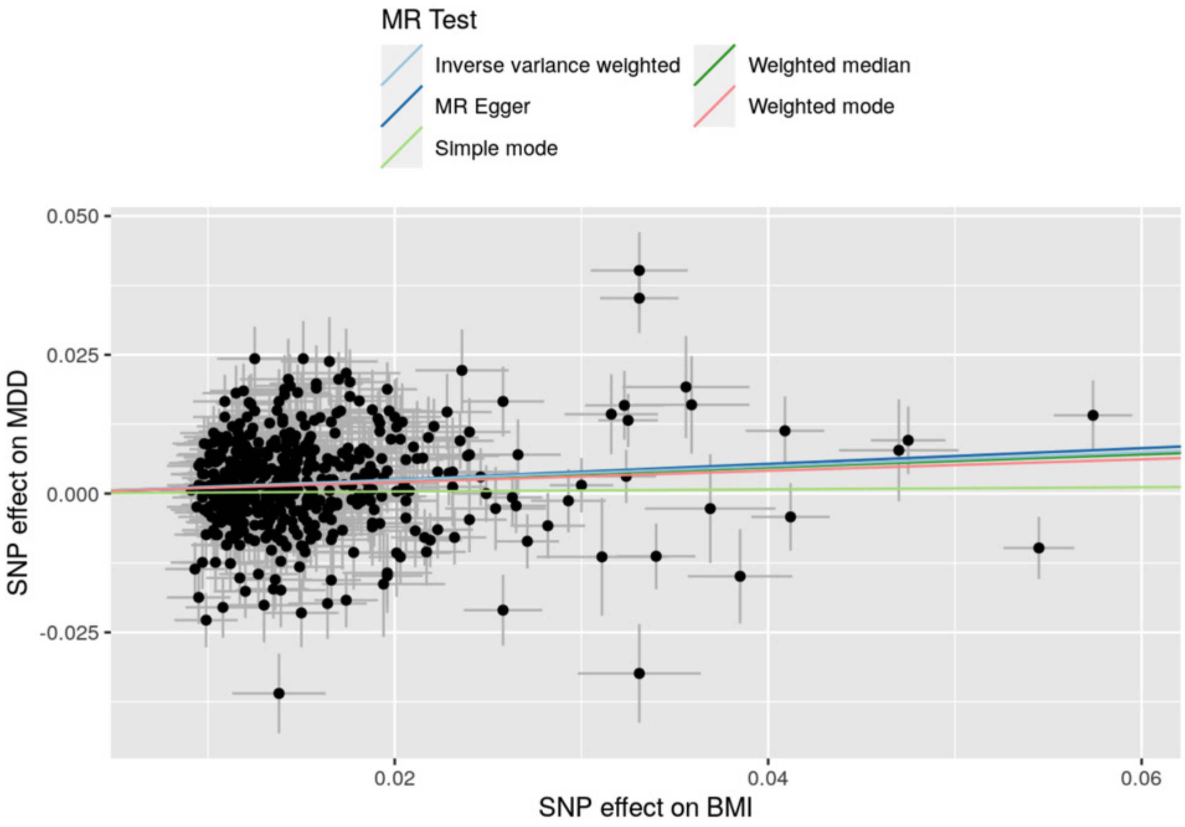

Figure S17. MR sensitivity analyses with BMI as exposure and MDD as the outcome

This figure illustrates the MR relationship between BMI and MDD. SNP effects associated with BMI are displayed on the x-axis and SNP effects associated with MDD are displayed on the y-axis. Each point represents the beta coefficient of these two traits. The horizontal line on each point represents the standard error associated with the exposure and the vertical line on each point represents the standard error associated with the outcome. Each coloured line represents a regression line using the MR test outlined in the legend key.

f. MDD on BMI

Table S9. Sensitivity analyses of MDD (exposure) on BMI (outcome)

exposure	outcome	method	nsnp	b	lo_ci	up_ci	pval
MDD	BMI	MR Egger	14	0.81737406	0.083769367	1.5509788	0.049554519
MDD	BMI	Weighted median	14	0.09889934	0.036993929	0.1608048	0.001740506
MDD	BMI	Inverse variance weighted	14	0.09814188	-0.051509285	0.2477931	0.198660758
MDD	BMI	Simple mode	14	0.08228897	-0.002303945	0.1668819	0.078914457
MDD	BMI	Weighted mode	14	0.08878637	0.012476840	0.1650959	0.040084366

Number of SNPS = nsnp / Beta = b / Lower 95% confidence interval = lo_ci / Upper 95% confidence interval = up_ci / P-value = pval

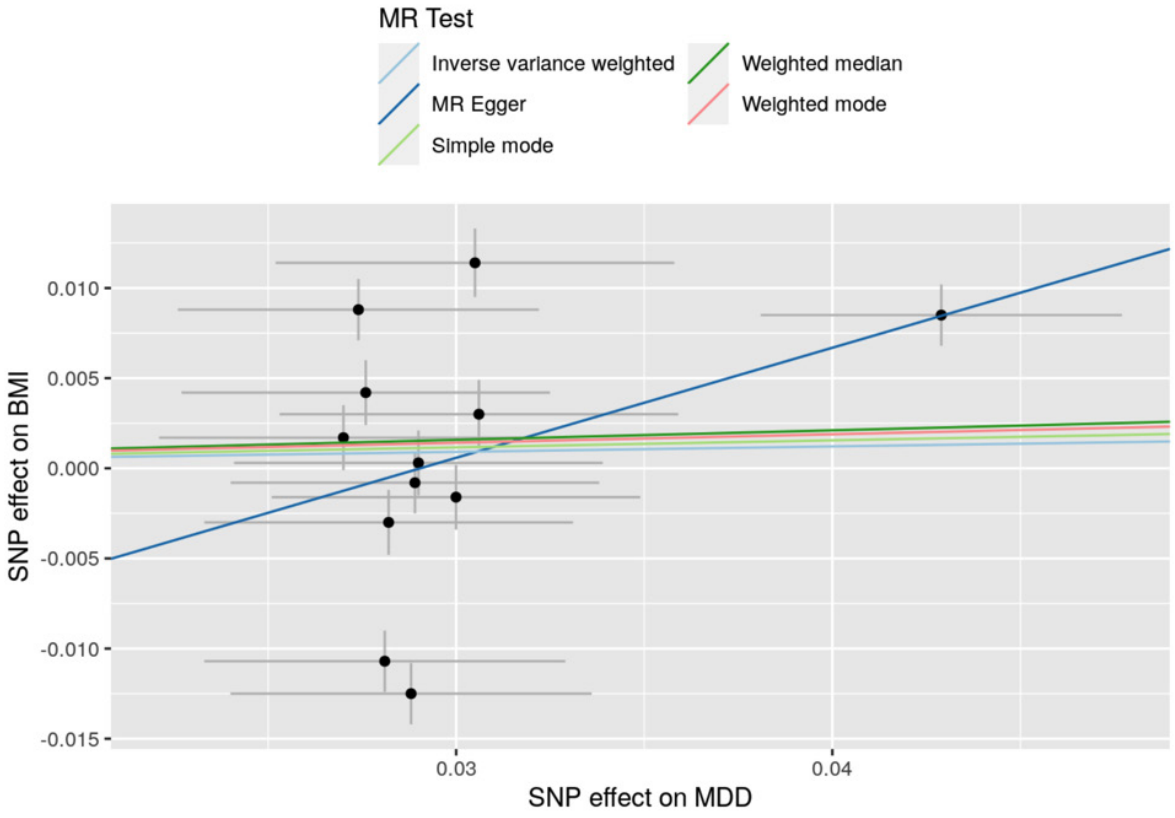

Figure S18. MR sensitivity analyses with MDD as exposure and BMI as the outcome

This figure illustrates the MR relationship between MDD and BMI. SNP effects associated with MDD are displayed on the x-axis and SNP effects associated with BMI are displayed on the y-axis. Each point represents the beta coefficient of these two traits. The horizontal line on each point represents the standard error associated with the exposure and the vertical line on each point represents the standard error associated with the outcome. Each coloured line represents a regression line using the MR test outlined in the legend key.

g. Childhood trauma on CRP

Table S10. Sensitivity analyses of childhood trauma (exposure) on CRP (outcome)

exposure	outcome	method	nsnp	b	lo_ci	up_ci	pval
TRAUMA	CRP	MR Egger	9	-1.2683595	-2.78004916	0.2433302	0.1440699
TRAUMA	CRP	Weighted median	9	0.1231611	-0.06401373	0.3103359	0.1971616
TRAUMA	CRP	Inverse variance weighted	9	0.1159220	-0.04389721	0.2757412	0.1551276
TRAUMA	CRP	Simple mode	9	0.1473378	-0.18304477	0.4777203	0.4075453
TRAUMA	CRP	Weighted mode	9	0.1115748	-0.17276499	0.3959145	0.4639314

Number of SNPS = nsnp / Beta = b / Lower 95% confidence interval = lo_ci / Upper 95% confidence interval = up_ci / P-value = pval

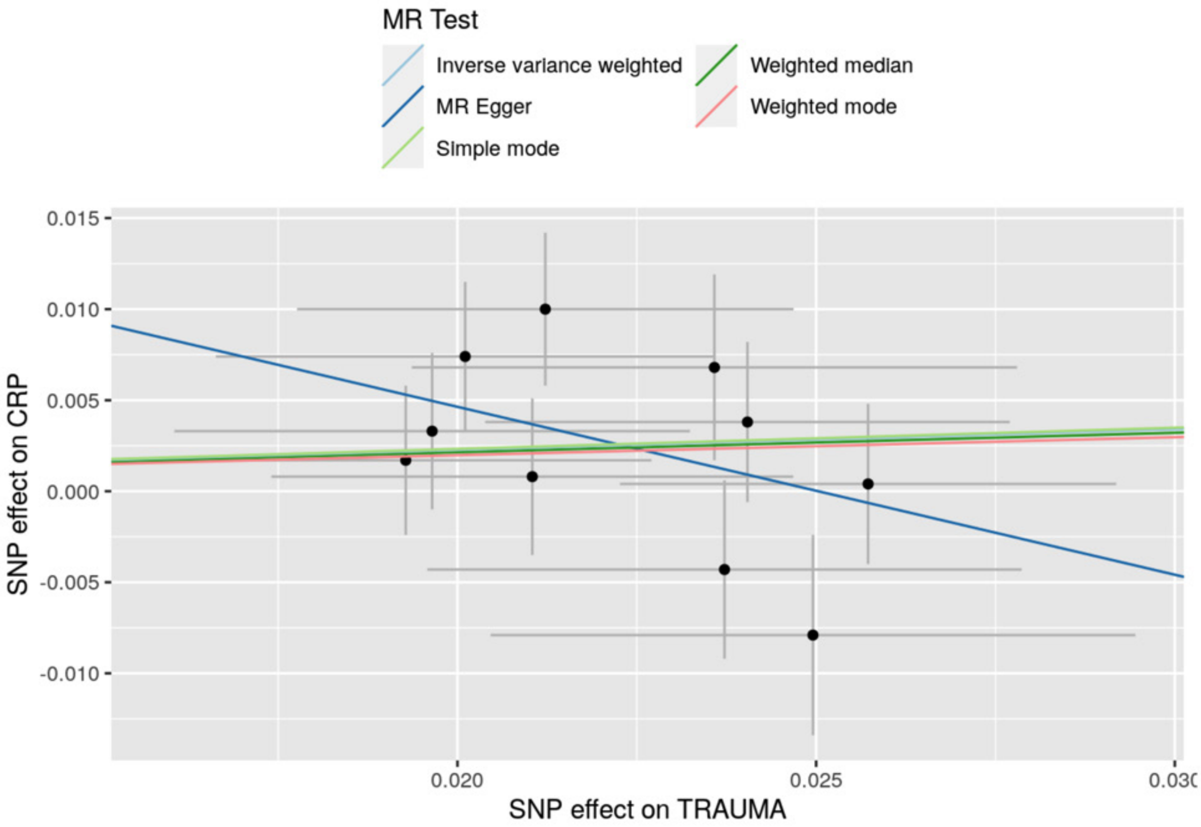

Figure S19. MR sensitivity analyses with childhood trauma as exposure and CRP as the outcome

This figure illustrates the MR relationship between childhood trauma and CRP. SNP effects associated with childhood trauma are displayed on the x-axis and SNP effects associated with CRP are displayed on the y-axis. Each point represents the beta coefficient of these two traits. The horizontal line on each point represents the standard error associated with the exposure and the vertical line on each point represents the standard error associated with the outcome. Each coloured line represents a regression line using the MR test outlined in the legend key.

h. CRP on childhood trauma

Table S11. Sensitivity analyses of CRP (exposure) on reported trauma (outcome)

exposure	outcome	method	nsnp	b	lo_ci	up_ci	pval
CRP	TRAUMA	MR Egger	122	0.01831330	-0.011053000	0.047679592	0.22399426
CRP	TRAUMA	Weighted median	122	0.01124354	-0.012678663	0.035165745	0.35694151
CRP	TRAUMA	Inverse variance weighted	122	0.01080567	-0.006179799	0.027791138	0.21243564
CRP	TRAUMA	Simple mode	122	-0.04742003	-0.096787024	0.001946963	0.06214237
CRP	TRAUMA	Weighted mode	122	0.01255569	-0.015424183	0.040535554	0.38085718

Number of SNPS = nsnp / Beta = b / Lower 95% confidence interval = lo_ci / Upper 95% confidence interval = up_ci / P-value = pval

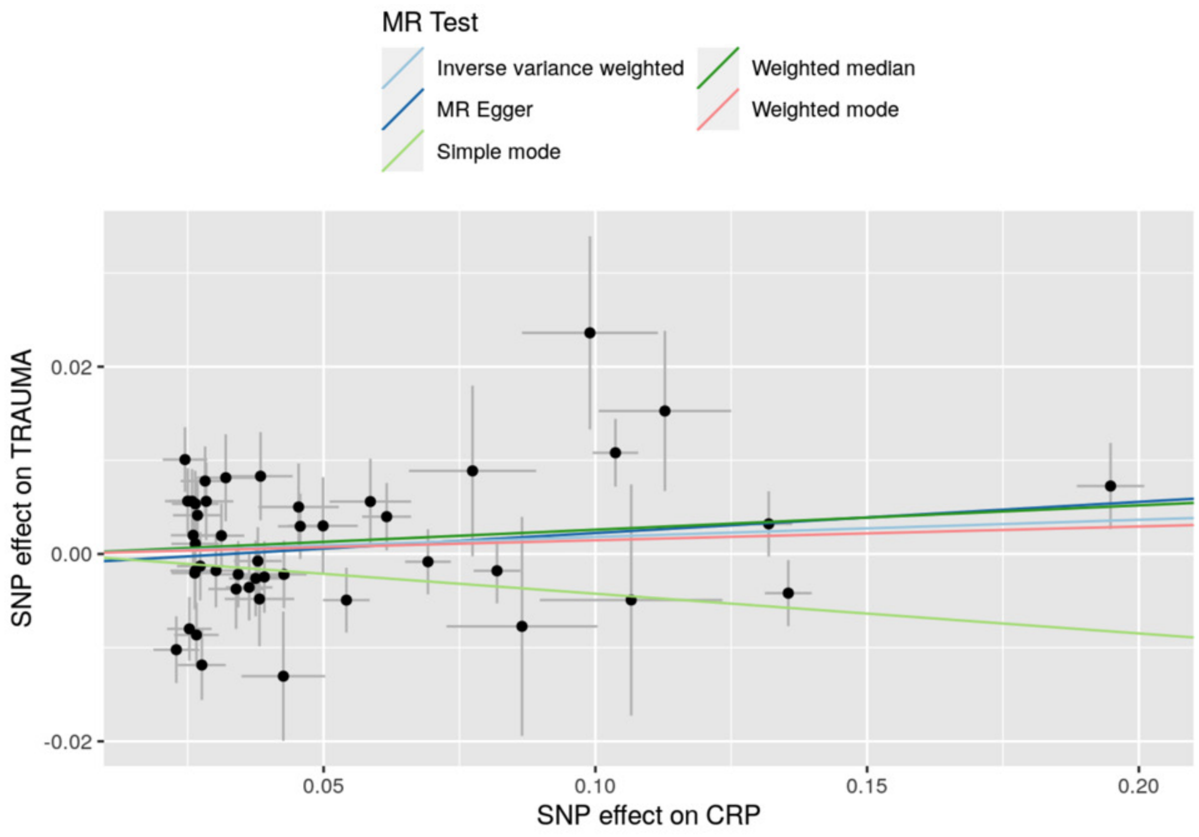

Figure S20. MR sensitivity analyses with CRP as exposure and childhood trauma as the outcome

This figure illustrates the MR relationship between CRP and childhood trauma. SNP effects associated with CRP are displayed on the x-axis and SNP effects associated with childhood trauma are displayed on the y-axis. Each point represents the beta coefficient of these two traits. The horizontal line on each point represents the standard error associated with the exposure and the vertical line on each point represents the standard error associated with the outcome. Each coloured line represents a regression line using the MR test outlined in the legend key.

i. Childhood trauma on MDD

Table S12. Sensitivity analyses of childhood trauma (exposure) on MDD (outcome)

exposure	outcome	method	nsnp	b	lo_ci	up_ci	pval
TRAUMA	MDD	MR Egger	9	-1.2459949	-3.7070130	1.2150233	3.540904e-01
TRAUMA	MDD	Weighted median	9	0.5079253	0.2729503	0.7429004	2.267654e-05
TRAUMA	MDD	Inverse variance weighted	9	0.5418212	0.2960481	0.7875942	1.553695e-05
TRAUMA	MDD	Simple mode	9	0.5957979	0.2007060	0.9908898	1.826650e-02
TRAUMA	MDD	Weighted mode	9	0.5703777	0.1622967	0.9784588	2.546869e-02

Number of SNPS = nsnp / Beta = b / Lower 95% confidence interval = lo_ci / Upper 95% confidence interval = up_ci / P-value = pval

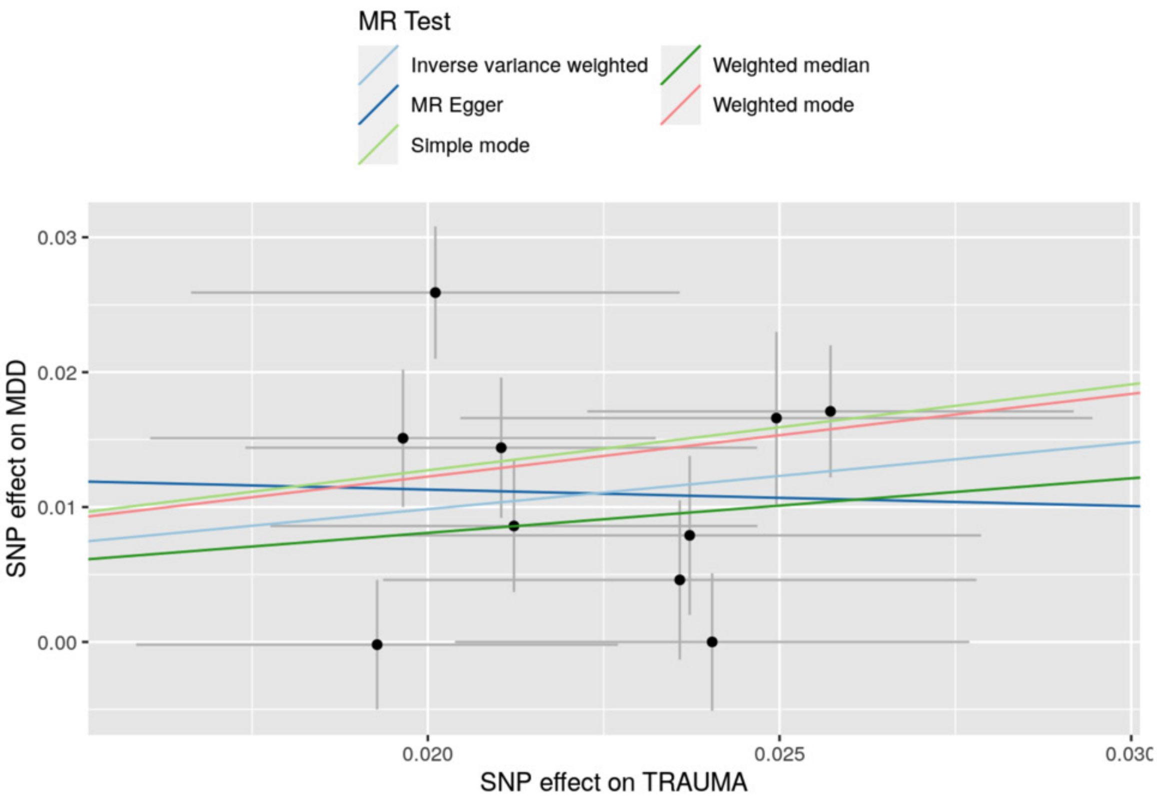

Figure S21. MR sensitivity analyses with childhood trauma as exposure and MDD as the outcome

This figure illustrates the MR relationship between childhood trauma and MDD. SNP effects associated with childhood trauma are displayed on the x-axis and SNP effects associated with MDD are displayed on the y-axis. Each point represents the beta coefficient of these two traits. The horizontal line on each point represents the standard error associated with the exposure and the vertical line on each point represents the standard error associated with the outcome. Each coloured line represents a regression line using the MR test outlined in the legend key.

j. MDD on childhood trauma

Table S13. Sensitivity analyses of MDD (exposure) on childhood trauma (outcome)

exposure	outcome	method	nsnp	b	lo_ci	up_ci	pval
MDD	TRAUMA	MR Egger	39	0.1671215	-0.19221832	0.5264613	3.679039e-01
MDD	TRAUMA	Weighted median	39	0.1490930	0.08848384	0.2097022	1.425400e-06
MDD	TRAUMA	Inverse variance weighted	39	0.1684021	0.10877681	0.2280274	3.099751e-08
MDD	TRAUMA	Simple mode	39	0.1522308	0.03004396	0.2744177	1.937338e-02
MDD	TRAUMA	Weighted mode	39	0.1601247	0.05074481	0.2695047	6.683095e-03

Number of SNPS = nsnp / Beta = b / Lower 95% confidence interval = lo_ci / Upper 95% confidence interval = up_ci / P-value = pval

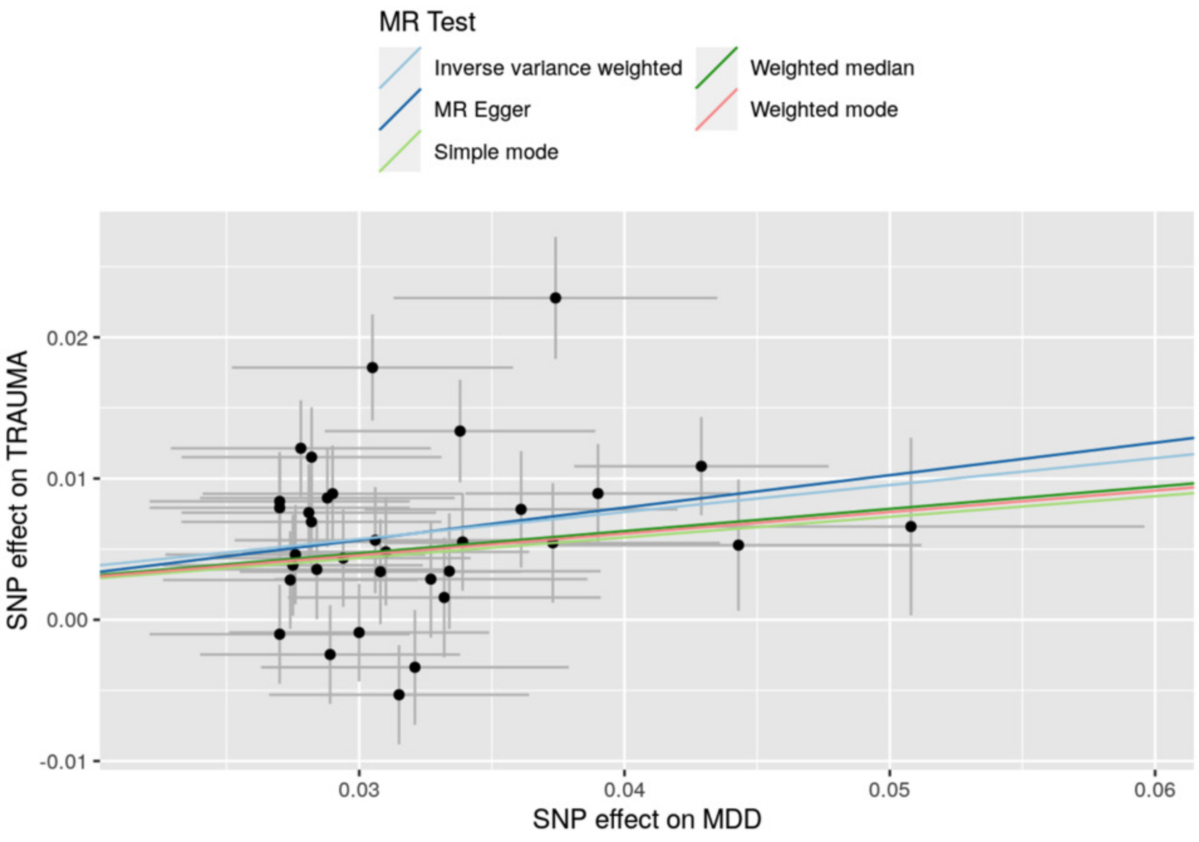

Figure S22. MR sensitivity analyses with MDD as exposure and childhood trauma as the outcome

This figure illustrates the MR relationship between MDD and childhood trauma. SNP effects associated with MDD are displayed on the x-axis and SNP effects associated with childhood trauma are displayed on the y-axis. Each point represents the beta coefficient of these two traits. The horizontal line on each point represents the standard error associated with the exposure and the vertical line on each point represents the standard error associated with the outcome. Each coloured line represents a regression line using the MR test outlined in the legend key.

k. BMI on childhood trauma

Table S14. Sensitivity analyses of BMI (exposure) on childhood trauma (outcome)

exposure	outcome	method	nsnp	b	lo_ci	up_ci	pval
BMI	TRAUMA	MR Egger	1210	0.07901132	0.01840696	0.1396157	1.073136e-02
BMI	TRAUMA	Weighted median	1210	0.08349578	0.05675121	0.1102404	9.412651e-10
BMI	TRAUMA	Inverse variance weighted	1210	0.09356167	0.07400228	0.1131211	6.878888e-21
BMI	TRAUMA	Simple mode	1210	0.06555600	-0.05154276	0.1826548	2.727389e-01
BMI	TRAUMA	Weighted mode	1210	0.07166605	-0.01817549	0.1615076	1.182014e-01

Number of SNPS = nsnp / Beta = b / Lower 95% confidence interval = lo_ci / Upper 95% confidence interval = up_ci / P-value = pval

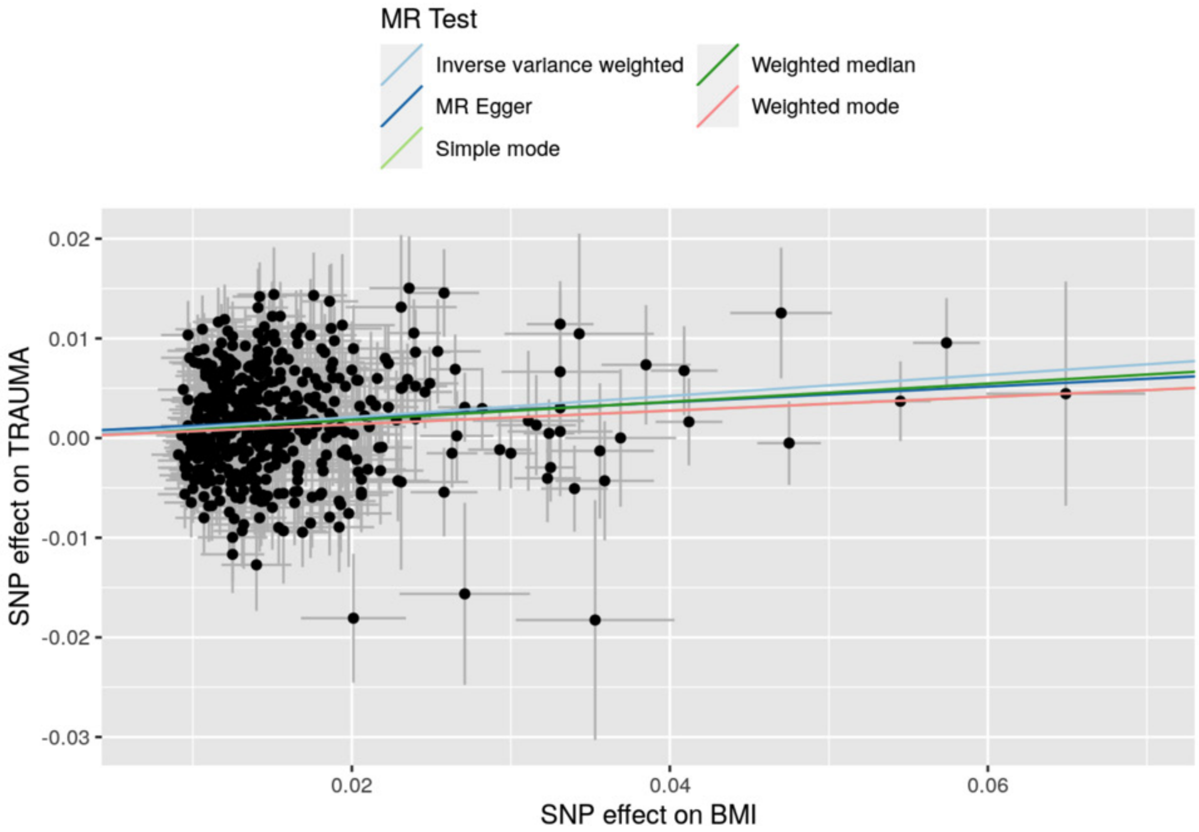

Figure S23. MR sensitivity analyses with BMI as exposure and childhood trauma as the outcome

This figure illustrates the MR relationship between BMI and childhood trauma. SNP effects associated with BMI are displayed on the x-axis and SNP effects associated with childhood trauma are displayed on the y-axis. Each point represents the beta coefficient of these two traits. The horizontal line on each point represents the standard error associated with the exposure and the vertical line on each point represents the standard error associated with the outcome. Each coloured line represents a regression line using the MR test outlined in the legend key.

l. Childhood trauma on BMI

Table S15. Sensitivity analyses of childhood trauma (exposure) on BMI (outcome)

exposure	outcome	method	nsnp	b	lo_ci	up_ci	pval
TRAUMA	BMI	MR Egger	3	-0.6942678	-2.12378733	0.7352518	0.515683610
TRAUMA	BMI	Weighted median	3	0.1853717	0.05111271	0.3196307	0.006806283
TRAUMA	BMI	Inverse variance weighted	3	0.2130452	0.03552759	0.3905627	0.018659434
TRAUMA	BMI	Simple mode	3	0.1868267	-0.01289239	0.3865457	0.208179557
TRAUMA	BMI	Weighted mode	3	0.1824019	0.02295570	0.3418481	0.154187208

Number of SNPS = nsnp / Beta = b / Lower 95% confidence interval = lo_ci / Upper 95% confidence interval = up_ci / P-value = pval

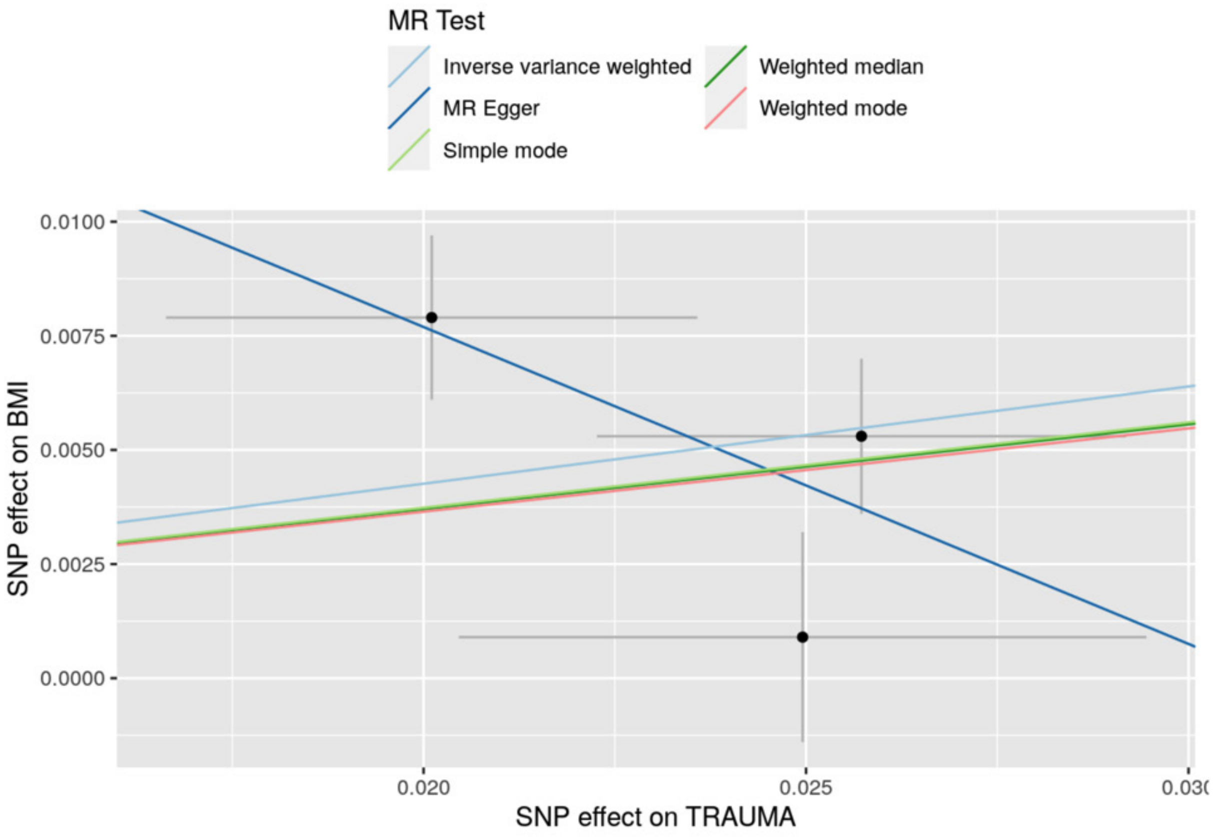

Figure S24. MR sensitivity analyses with childhood trauma as exposure and BMI as the outcome

This figure illustrates the MR relationship between childhood trauma and BMI. SNP effects associated with childhood trauma are displayed on the x-axis and SNP effects associated with BMI are displayed on the y-axis. Each point represents the beta coefficient of these two traits. The horizontal line on each point represents the standard error associated with the exposure and the vertical line on each point represents the standard error associated with the outcome. Each coloured line represents a regression line using the MR test outlined in the legend key.

9. mtCOJO sensitivity analyses results

Given that BMI is known to be a strong confounding factor in this model, we conditioned the other three traits (MDD, CRP and childhood trauma) on BMI and performed the same MR analyses using GSMR. Our results show that when conditioned on BMI, childhood trauma is genetically predicting an increased risk of MDD (OR: 1.89, 95% CI: 1.62 – 2.20, p = < 0.001) whereby childhood trauma is associated with a 89% higher odds of MDD; MDD is genetically predicting childhood trauma (OR: 1.22, 95% CI: 1.16 – 1.27, p = < 0.001); and MDD is associated with a decrease in BMI (β: -0.11, 95% CI: -0.09 to -0.14, p = < 0.001) whereby MDD results in a 0.11 kg/m^2 decrease in BMI. These results remain significant after Bonferroni correction ($p_{Bonferroni}$ = 0.004).

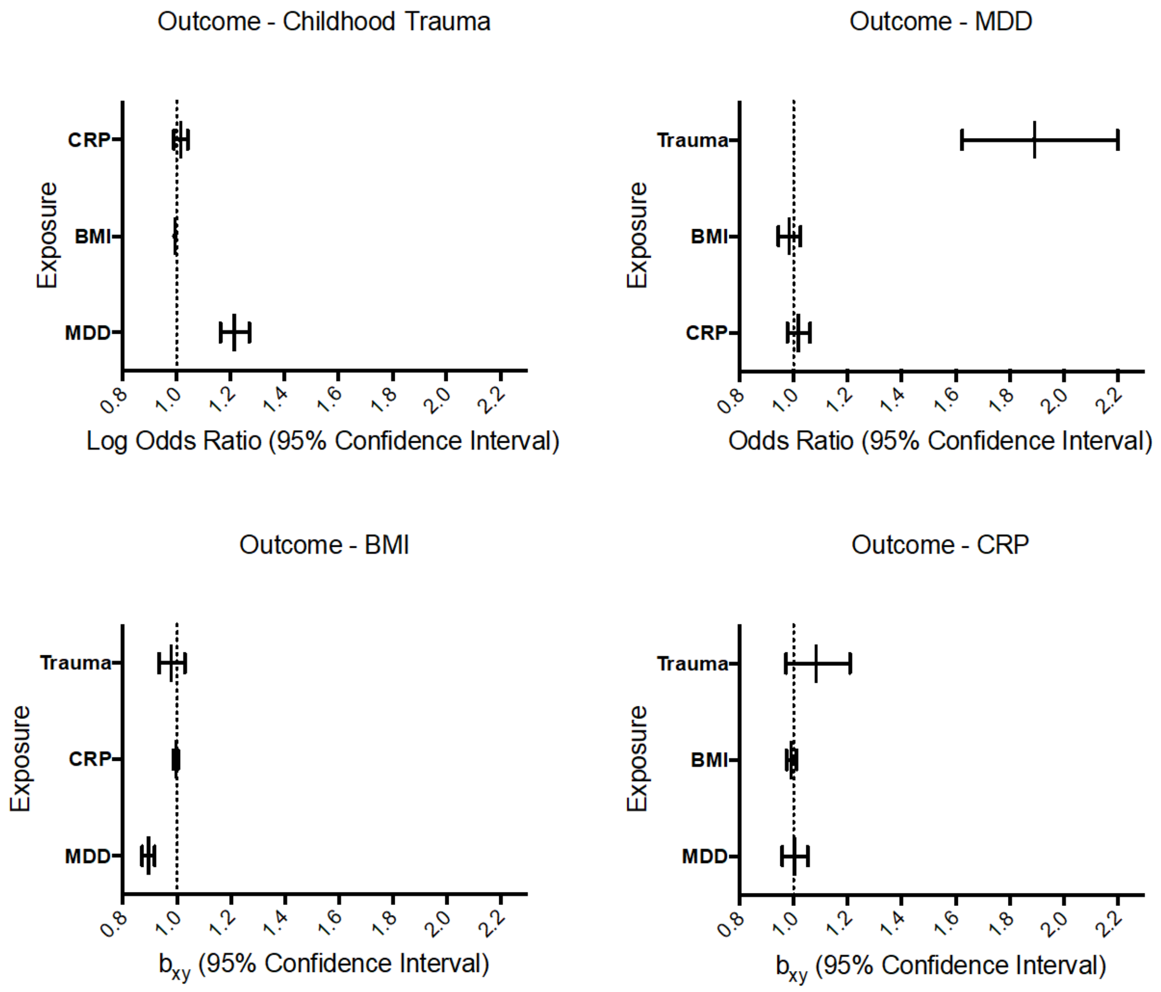

Figure S25. All generalised-summary mendelian randomization (GSMR) results conditioned on BMI using mtCOJO

(BMI = Body Mass Index; CRP = C-Reactive Protein; MDD = Major Depressive Disorder)

This figure represents a summary of three bi-directional GSMR analyses involving four traits (12 analyses in total). The charts are split by the exposure of interest. Dots represent effect sizes (as measured by odds ratios, ORs) on the liability scale of the disorders of risk factors on traits (childhood trauma and MDD) and effect sizes (as measured by β, b_{xy}) on the liability scale of the disorders of risk factors on traits (BMI and CRP). Each outcome is labelled on the y-axis and the strength of each exposure on the outcome displayed on the x-axis (as an odds ratio or a beta, plotted on a linear scale). Error bars represent 95% confidence intervals. Childhood trauma was associated with increased odds of MDD, and MDD was associated with a decrease in BMI and an increased odds of childhood trauma, after multiple testing correction ($p_{Bonferroni}$ = 0.004).

10. cis-CRP SNP effects on MDD and childhood trauma

Our findings are mixed, suggesting that cis-CRP genetic variants mildly genetically predict higher odds of MDD at R2 < 0.8 (OR: 1.03, 95% CI: 1.00 – 1.05, p = 0.02), although they do not at the advised threshold of R2 < 0.05 (OR: 1.02, 95% CI: 0.97 – 1.06, p = 0.5). They also show that cis-CRP genetic variants predict lower odds of childhood trauma at R2 < 0.2 (OR: 0.97, 95% CI: 0.95 – 1.00, p = 0.04), R2 < 0.4 (OR: 0.97, 95% CI: 0.95 – 0.99, p = 0.01), R2 < 0.6 (OR: 0.97, 95% CI: 0.95 – 0.99, p = 0.01) and R2 < 0.8 (OR: 0.97, 95% CI: 0.95 – 0.99, p < 0.01), although they do not at the advised R2 < 0.05 (OR: 0.97, 95% CI: 0.94 – 1.00, p = 0.06).

Table S16. GSMR output with cis-CRP SNPs only, at different clumping thresholds

R2	Exposure	Outcome	Beta xy	Lower CI	Upper CI	P	N SNPS
< 0.05	CRP	MDD	0.015	-0.02812	0.05812	0.5	3
< 0.2	CRP	MDD	0.023	-0.01424	0.06024	0.219	7
< 0.4	CRP	MDD	0.023	-0.00836	0.05436	0.144	12
< 0.6	CRP	MDD	0.024	-0.0054	0.0534	0.117	12
< 0.8	CRP	MDD	0.029	0.00548	0.05252	0.019	17

R2	Exposure	Outcome	Beta xy	Lower CI	Lower CI	P	N SNPS
< 0.05	CRP	Trauma	-0.031	-0.06236	0.00036	0.057	3
< 0.2	CRP	Trauma	-0.028	-0.05348	-0.00252	0.036	7
< 0.4	CRP	Trauma	-0.027	-0.04856	-0.00544	0.014	12
< 0.6	CRP	Trauma	-0.029	-0.05056	-0.00744	0.009	12
< 0.8	CRP	Trauma	-0.032	-0.04964	-0.01436	0	17

Note: Each row represents one Mendelian randomization test between the exposure and the outcome. MDD = major depressive disorder; CRP = C-reactive protein; R2 = the clumping threshold for SNPs; Beta xy = beta of the exposure (x) on the outcome (y); Lower CI = lower 95% confidence interval; Upper CI = upper 95% confidence interval;; P = p-value; nSNP = number of SNPs used in the analyses.

11. Multivariable MR analyses of all traits on MDD

Table S17. Multivariable MR output with BMI, CRP and reported trauma as joint exposures, and MDD as the outcome

This table displays the results from Multivariable MR analyses, which model the joint effects of exposures (reported trauma, CRP and BMI) on a single outcome (MDD). Therefore, outcomes for each exposure display the direct effects on MDD, whilst accounting for the other two exposures.

Exposure	Beta	SE	T-value	P	OR	Lower CI	Upper CI
Reported Trauma	0.45	0.00	99.20	0.00E+00	1.57	1.56	1.58
CRP	-0.02	0.00	-6.67	2.61E-11	0.98	0.98	0.99
BMI	0.10	0.00	43.37	0.00E+00	1.11	1.10	1.12

Note: these results represent the joint exposure effects of BMI, CRP and reported trauma on MDD (outcome). CRP = C-reactive protein; BMI = body mass index; SE = standard error; P = p-value; OR = odds ratio; CI = confidence interval.

12. Testing the strength of our genetic instruments

Table S18. F-statistic and I-squared statistic tests of our genetic instruments

This table displays results of F-statistic tests, as a measure of instrument strength; and the adapted I-squared statistic measure as an indicator of the strength of the NOME violation for MR Egger (12). Results from the F-statistic test indicates relative strength of all genetic instruments (> 30). However, the I-squared statistic is below the acceptable threshold for reported trauma and MDD, indicating that MR egger estimates when these two traits are the exposures of interest should be interpreted with caution.

	**Trauma**	**CRP**	**BMI**	**MDD**
F-statistic	34.02	104.22	57.03	34.89
I-squared statistic	0.00	0.97	0.85	0.12

Note: these results represent the F-statistic and I-squared statistic test results, for each of our genetic instruments. CRP = C-reactive protein; BMI = body mass index; MDD = major depressive disorder.

13. Testing for shared or causal effects between CRP, reported trauma, BMI and MDD using MR-CAUSE

a) CRP on MDD

Table S19. The effects of CRP on MDD using MR-CAUSE

In this case we see that neither model is significant.

	model1	model2	delta_elpd	se_delta_elpd	z	p
1	null	sharing	0.22	0.35	0.62	0.73
2	null	causal	-0.86	1.80	-0.48	0.32
3	sharing	causal	-1.10	1.50	-0.73	0.23

Note: delta_elpd = delta expected log pointwise posterior density (the estimator) – if the delta_elpd is negative, model 2 is a better fit; se_delta_elpd = standard error of the estimator; z = z-score that can be compared to a normal distribution to test if the difference in model fit it significant; p = corresponding one-sided p-value.

b) MDD on CRP

In this case we see that neither model is significant.

	model1	model2	delta_elpd	se_delta_elpd	z	p
1	null	sharing	0.095	0.62	0.15	0.56
2	null	causal	0.170	1.60	0.11	0.54
3	sharing	causal	0.079	0.98	0.08	0.53

Note: delta_elpd = delta expected log pointwise posterior density (the estimator) – if the delta_elpd is negative, model 2 is a better fit; se_delta_elpd = standard error of the estimator; z = z-score that can be compared to a normal distribution to test if the difference in model fit it significant; p = corresponding one-sided p-value.

c) Reported trauma on MDD

In this case we see that the sharing model, with the lowest delta_elpd and the smallest standard error is the best fit.

	model1	model2	delta_elpd	se_delta_elpd	z	p
1	null	sharing	-2.4	1.4	-1.7	0.043
2	null	causal	-6.3	3.3	-1.9	0.029
3	sharing	causal	-3.9	2.0	-2.0	0.023

Note: delta_elpd = delta expected log pointwise posterior density (the estimator) – if the delta_elpd is negative, model 2 is a better fit; se_delta_elpd = standard error of the estimator; z = z-score that can be compared to a normal distribution to test if the difference in model fit it significant; p = corresponding one-sided p-value.

d) MDD on Reported trauma

In this case we see that the causal model with the lowest delta_elpd and the lowest standard error is the best fit.

	model1	model2	delta_elpd	se_delta_elpd	z	p
1	null	sharing	-5.5	2.6	-2.1	0.019
2	null	causal	-9.2	4.2	-2.2	0.015
3	sharing	causal	-3.7	1.7	-2.2	0.013

Note: delta_elpd = delta expected log pointwise posterior density (the estimator) – if the delta_elpd is negative, model 2 is a better fit; se_delta_elpd = standard error of the estimator; z = z-score that can be compared to a normal distribution to test if the difference in model fit it significant; p = corresponding one-sided p-value.

e) BMI on MDD

In this case we see that the sharing model with the lowest delta_elpd and the lowest standard error is the best fit.

	model1	model2	delta_elpd	se_delta_elpd	z	p
1	null	sharing	-2.4	1.4	-1.7	0.043
2	null	causal	-6.3	3.3	-1.9	0.029
3	sharing	causal	-3.9	2.0	-2.0	0.023

Note: delta_elpd = delta expected log pointwise posterior density (the estimator) – if the delta_elpd is negative, model 2 is a better fit; se_delta_elpd = standard error of the estimator; z = z-score that can be compared to a normal distribution to test if the difference in model fit it significant; p = corresponding one-sided p-value.

f) MDD on BMI

In this case we see that the causal model with the lowest delta_elpd and the lowest standard error is the best fit.

	model1	model2	delta_elpd	se_delta_elpd	z	p
1	null	sharing	-5.2	2.5	-2.1	0.018
2	null	causal	-9.2	4.2	-2.2	0.015
3	sharing	causal	-3.9	1.8	-2.2	0.014

Note: delta_elpd = delta expected log pointwise posterior density (the estimator) – if the delta_elpd is negative, model 2 is a better fit; se_delta_elpd = standard error of the estimator; z = z-score that can be compared to a normal distribution to test if the difference in model fit it significant; p = corresponding one-sided p-value.

14. Supplementary References

1. Kessler RC, Ustün TB: The World Mental Health (WMH) Survey Initiative Version of the World Health Organization (WHO) Composite International Diagnostic Interview (CIDI). Int J Methods Psychiatr Res 2004; 13:93–121
2. Palmos AB, Watson S, Hughes T, et al.: Associations between childhood maltreatment and inflammatory markers [Internet]. BJPsych Open 2019; 5Available from: http://dx.doi.org/10.1192/bjo.2018.80
3. Palmos AB, Chung R, Frissa S, et al.: Reconsidering the reasons for heightened inflammation in major depressive disorder [Internet]. J Affect Disord 2020; Available from: http://www.sciencedirect.com/science/article/pii/S0165032720331992
4. Bycroft C, Freeman C, Petkova D, et al.: The UK Biobank resource with deep phenotyping and genomic data. Nature 2018; 562:203–209
5. McCarthy S, Das S, Kretzschmar W, et al.: A reference panel of 64,976 haplotypes for genotype imputation. Nat Genet 2016; 48:1279–1283
6. Coleman JRI, Euesden J, Patel H, et al.: Quality control, imputation and analysis of genome-wide genotyping data from the Illumina HumanCoreExome microarray. Brief Funct Genomics 2016; 15:298–304
7. Choi SW, O'Reilly PF: PRSice-2: Polygenic Risk Score software for biobank-scale data [Internet]. Gigascience 2019; 8Available from: http://dx.doi.org/10.1093/gigascience/giz082
8. Wray NR, Ripke S, Mattheisen M, et al.: Genome-wide association analyses identify 44 risk variants and refine the genetic architecture of major depression. Nat Genet 2018; 50:668–681
9. Yengo L, Sidorenko J, Kemper KE, et al.: Meta-analysis of genome-wide association studies for height and body mass index in ∼700000 individuals of European ancestry. Hum Mol Genet 2018; 27:3641–3649
10. Ligthart S, Vaez A, Võsa U, et al.: Genome Analyses of >200,000 Individuals Identify 58 Loci for Chronic Inflammation and Highlight Pathways that Link Inflammation and Complex Disorders. Am J Hum Genet 2018; 103:691–706
11. Warrier V, Kwong ASF, Luo M, et al.: Gene-environment correlations and causal effects of childhood maltreatment on physical and mental health: a genetically informed approach. Lancet Psychiatry 2021; 8:373–386
12. Bowden J, Del Greco M F, Minelli C, et al.: Assessing the suitability of summary data for two-sample Mendelian randomization analyses using MR-Egger regression: the role of the I2 statistic. Int J Epidemiol 2016; 45:1961–1974
